# Weed detection in blackgram fields under occlusion and class imbalance using SA–ANMS Faster R-CNN

**DOI:** 10.1038/s41598-026-52802-2

**Published:** 2026-05-12

**Authors:** Deepthi G Pai, Mamatha Balachandra, Radhika Kamath

**Affiliations:** Manipal Institute of Technology, Manipal Academy of Higher Education, Manipal, 576104 India

**Keywords:** Attention mechanism, Crop-weed detection, Deep learning, Faster R-CNN, Multiscale fusion, Object detection, Precision agriculture, Engineering, Mathematics and computing, Plant sciences

## Abstract

Sustainable agriculture has been greatly challenged by the problems of dense canopies that result in extreme occlusion, scale disparities and imbalances of classes, making it difficult to detect small and overlapping weeds in real field conditions. Actual blackgram fields in Karnataka, India, were used to generate a dataset that featured a high level of crop to weed contest, and exhibited a significant amount of scale diversity, with 75 percent of objects comprising less than 0.8 percent of the image space. To address these challenges, the paper presents a better Faster R-CNN framework that adds five modules, i.e. Spatial Attention (SA), Multi-Scale Fusion (MSF), Context-Aware RoI, Shape-Aware Prediction and Adaptive Non-Maximum Suppression (ANMS). The research shows that with larger architectural complexity, there is no guarantee of better performance through an extensive ablation study involving 32 model configurations. The best two-module combination (SA + ANMS) obtained the highest F1 -score of 0.9547, precision of 0.9439, recall of 0.9658, and a mean Intersection over Union (mIoU) of 0.9445, and inference speed of 4.42 FPS, which was higher than the full five-module version. It is important to note that only 12.5% of configurations were better than the baseline, highlighting the fact that too much module stacking may hurt performance. The analysis shows that there is a high positive synergy between SA and ANMS and that the MSF module competes slightly on features. The novelty of this work lies in the systematic exploration of how various enhancement modules can be interacted to function as a single detection framework and show that the selective combination of modules can produce a better outcome than random stacking. Especially, the finding of a complementarity, optimum minimum combination of SA + ANMS confirms that the efficiency-based design is used, not complexity-based expansion. It provides informative insight on designing efficient detection system for precision agriculture applications by revealing the impact of wise module selection instead of architecture complexity on obtaining consistent weed detection against occlusion and inter-class inconsistencies.

## Introduction

The world’s economy is based on agriculture, but because of resource limitations and population increases, there exists a challenge in the field. Data technology is helping the sector maximize productivity while minimizing negative externalities^1^. Artificial Intelligence has made tremendous but steady improvements over the last 50 years and has been successfully employed, especially in the agricultural sector. Crop disease infestations, storage management issues, pesticide control issues, and weed control are just a few among the numerous challenges faced by farmers^2^.

Pulses, known as the ”poor man’s protein source” with a protein concentration of 20 to 25%, are vital for vegetarian diets and soil health^3–6^. Global pulse production has grown at an annual rate of 1.3%, yet demand, especially in India, is exceeding supply, necessitating a production increase of 2.2% to meet anticipated needs of 39 million metric tons annually by 2050^7^. Pulses represent a significant crop in India’s agriculture, cultivated over 28.78 million hectares with a yield of 25.46 million tonnes. India, producing 70% of the world’s blackgram (*Vigna mungo* L.), is critical as blackgram is rich in protein (22.3%) and carbohydrates (48.0%), alongside essential minerals such as calcium and iron^8^. But blackgram production is severely affected by the weed presence.

Farmers have to invest heavily in the actual management of the lands as the weeds spread quickly, affecting the production of crops through yield suppression. There are five recognized forms of weed management: the chemical method, the mechanical method, the biological method, the cultural method, and the preventative method. Although these methods have yielded acceptable results, the downside to these methods is the fact that they can prove too costly, time-consuming, and have the potential to poison the environment as well as human health. There have been several methods of detecting the presence of weeds, including the use of several tools^9,10^. The analysis of the detection of weeds in the crops has persisted in the application of Artificial Intelligence techniques like Machine Learning (ML) and Deep Learning (DL)^11^.

Crop–weed competition imposes physiological stress on cultivated plants that extends beyond resource competition. Recent studies have demonstrated that weed proximity triggers ROS/RNS-mediated redox signaling cascades in crop species, which modulate stress adaptation responses at the molecular level^12^. Thus, early and accurate detection of weeds in field imagery, as tackled by this study, gives a crucial intervention opportunity before those biochemical stresses occur and this emphasizes the agronomical necessity of automated weed detection systems.

Applications of deep learning for weed detection have surged since 2015, dominated by Convolutional Neural Networks (CNNs) and their variants such as SegNet, GoogLeNet, ResNet, DetectNet and VGGNet, demonstrating that Deep Learning is effective for complex image recognition^11,13,14^. High precision in weed/crop discrimination is needed for targeted herbicide application^15^ with the use of deep learning, with potential identified in the Faster R-CNN model^16^, however issues remain for small objects and tightly grouped instances.

The present work proposes the SA-ANMS model, an improvement of the Faster R-CNN model, for overcoming issues of class imbalance, occlusion, and dominance during blackgram crop and weed detection. It has five improvement modules; Spatial Attention(SA), Multi-Scale Fusion(MSF), Context-Aware RoI(CR), Shape-Aware Prediction(SP), and Adaptive Non-Maximum Suppression(ANMS), resulting in thirty-two combinations of architecture. The evaluation for each combination was mainly based on F1-score on the imbalanced datasets along with precision and recall values. It was found that the combination of Spatial Attention module and Adaptive Non-Maximum Suppression module resulted in an F1-score of 95.47, and also decreased the computation overhead. Hence, the best model was chosen for better efficiency in crop detection.

## Related works

Faster R-CNN is a popular object detection model for weed detection as it yields a high localization accuracy. The RPN allows for end-to-end training and an increased quality of proposals, sharing convolutional features to boost the detection performance on benchmarks like PASCAL VOC and MS COCO^16^. However the computational burden of Faster R-CNN makes it not applicable to real time field application.

Many studies have been proposed to optimize Faster R-CNN in agriculture environments through improving backbone and feature enhancement. Among these methods, combining Feature Pyramid Networks and ResNeXt backbones enables multi-scale features extraction and has obtained competitive accuracy for complex background^17^. CBAM with VGG19 on the contrary enhances recognition results of soybean fields by attention mechanism^18^. Although these methods effectively improve feature representation, they largely focus on high accuracy and can neglect severe occlusion and dense overlap objects that frequently appear in actual crop fields.

Comparison between one stage and two stage detectors have revealed an accuracy-speed trade-off. One-stage models like YOLO based models with newer version YOLOv8-YOLOv11 achieves faster inference and comparable accuracy as compared to the best two-stage model Faster R-CNN which is better in localization^19^. Nevertheless, both families of model suffers from the small object detection and class imbalance issues. In dense agricultural scene these problem becomes more severe. Comparisons among UAV based approaches and backbones show improvement only for specific dataset, does not show any generalization performance for different field conditions^20,21^.

In recent years, weed detection has undergone a few changes that mostly involved architectural developments such as feature fusion, transformer-based networks, and hybrid networks. Among them, some feature fusion based methods (PD-YOLO), utilize adaptive feature pyramid and attention module to enhance detection robustness^22^, while transformer based networks (WeedSwin), reach incredible high accuracy on large datasets^23^. Light and efficient models have also been designed due to the restricted hardware conditions^24,25^. However, most of the above methods either focus on calculation efficiency or on detection accuracy, and pays little attention on modules combination effect or the accuracy versus efficiency tradeoff in complex real field.

Additionally, other newly developed techniques like knowledge distillation and spatio-temporal analysis also enhance performance and robustness^26,27^, but they can only be applied when a large dataset or controlled environment is available. It becomes clear that a significant shortcoming is the absence of a detailed study on how various architectural components behave under occlusions, scale variance and class imbalance.

To compensate the above drawbacks, this paper, systematically research the combination of various enhancement modules into Faster R-CNN framework, particularly on their interactions, performance trade-offs and robustness on practical field scenarios involving occlusion, scale variability and class imbalance.

## Research gaps identified

Despite the great progress in Deep Learning-based weed detection, there are still several key limitations in existing studies. These limitations motivated the development of the proposed SA-ANMS.

- Limited robustness under severe occlusion and dense canopy conditions: Many Faster R-CNN and YOLO based weed detection models suffer from poor performance due to occluded weeds in dense crop environments, which reduces detection reliability. In this work, a Spatial Attention (SA) mechanism is proposed to be integrated to enhance feature learning and localization of partially visible weeds.
- Inadequate handling of extreme class imbalance: Weed datasets from real fields usually have a highly imbalanced class distribution, i.e. weed classes with few occurrences are underrepresented. Traditional methods typically rely on fixed non-maximum suppression thresholds that may suppress true detections of minority classes. The proposed adaptive non-maximum suppression (ANMS) introduces class-specific thresholds for preserving detections in minority and densely distributed classes.
- Challenges in detecting small and overlapping weed instances: Traditional approaches in agriculture experience difficulty in maintaining close proximity detections of weed objects and overlapping structures because of consistent suppression processes. The proposed technique improves the conservation of such detections using an adaptive suppression approach.
- Overemphasis on controlled datasets rather than real-field variability: Several studies assess models with controlled datasets, limiting their generalization in real agricultural contexts.The SA-ANMS framework is presented herein with results generated from real-field blackgram data with characteristics including occlusion, density, and variation.
- The poor integration of feature enhancement and detection refinement approaches: Though majority of attention-based weed detection approaches focus on feature enhancement, post-processing stage is often neglected. SA-ANMS framework closes this gap and utilizes spatial attention and adaptive suppression to achieve a richer feature representation and improved prediction.

## Research objectives

This work introduces an improved object detection architecture which is especially adapted to the problem of crop weed discrimination with following aims:

1. Formulate an improved Faster R-CNN based architecture to perform the detection of crop and weeds in blackgram fields facing dense occlusion and highly imbalanced classes.
2. To comprehensively evaluate the performance enhancement obtained using the five architectural modules viz Spatial Attention,Multi Scale Fusion, Context Aware RoI, Shape aware Prediction and Adaptive Non Maximum suppression individually and their combination.
3. Determine the architecture composition achieving best accuracy (measured through F1-score) and computational cost whichfurther led to the selection of the proposed model SA- ANMS.

### Contributions and novelty

The novel aspects of this research can be summarized as:

- Development of the real-field dataset from Karnataka, India, of blackgram fields, that includes significant class imbalance,highly occluded, and tiny weeds, 75% of which are less than 0.8% of image bounding box area. The extreme variation in scales of the fields is also reflected in the variation in the sizes ranging from 7995 to 8,165,598 *px*^2^ that requires the appropriate architecture.
- Lightweight SA (spatial attention) module (1–2% of computational overhead) that captures crop-weed relationship and helpsin detection of small objects in dense fields.
- Memory-efficient Multi-Scale Fusion (MSF) module to handle extreme scale variations and enhance detection of tiny weeds.
- Context-Aware RoI (CR) head incorporating global scene information to improve localization in highly occluded and overlap-ping regions.
- Shape-Aware Prediction (SP) box predictor with an auxiliary shape learning branch to better capture irregular weed and crop shapes.
- Adaptive Non-Maximum Suppression (ANMS) mechanism to reduce false positives in crowded field scenarios.
- A novel SA-ANMS Model is proposed, derived from the Faster R-CNN baseline through selective architectural enhancement,specifically designed to address severe class imbalance, dense canopy occlusion, and extreme scale variation in real-field crop–weed detection.

## Methodology

### Image acquisition

Images involving blackgram crops (*Vigna mungo*) and weeds were collected from fields in Dharwad, Karnataka, India—an area with semi-arid climatic conditions ideal for the growth of blackgram. Images from blackgram fields were acquired during the critical growth stage characterized by intense crop–weed competition. For increased diversity in the dataset and to increase model generalization, images were taken from different heights and angles. The dataset comprises different categories of weed species, such as more than 12 broadleaved varieties and different types of grassy weeds which include grasses and sedges. These collected images of weeds show various conditions found in fields, such as dense grouping of weeds, overlap between crops and weeds, and natural background complexity. Challenging examples have also been added in the dataset, such as partially buried weeds in soil, very young stage weeds, and crops and weeds with high visual similarity. This dataset ensures that it encompasses real-world conditions for agriculture and helps to build a robust detection models.

**Fig. 1:**
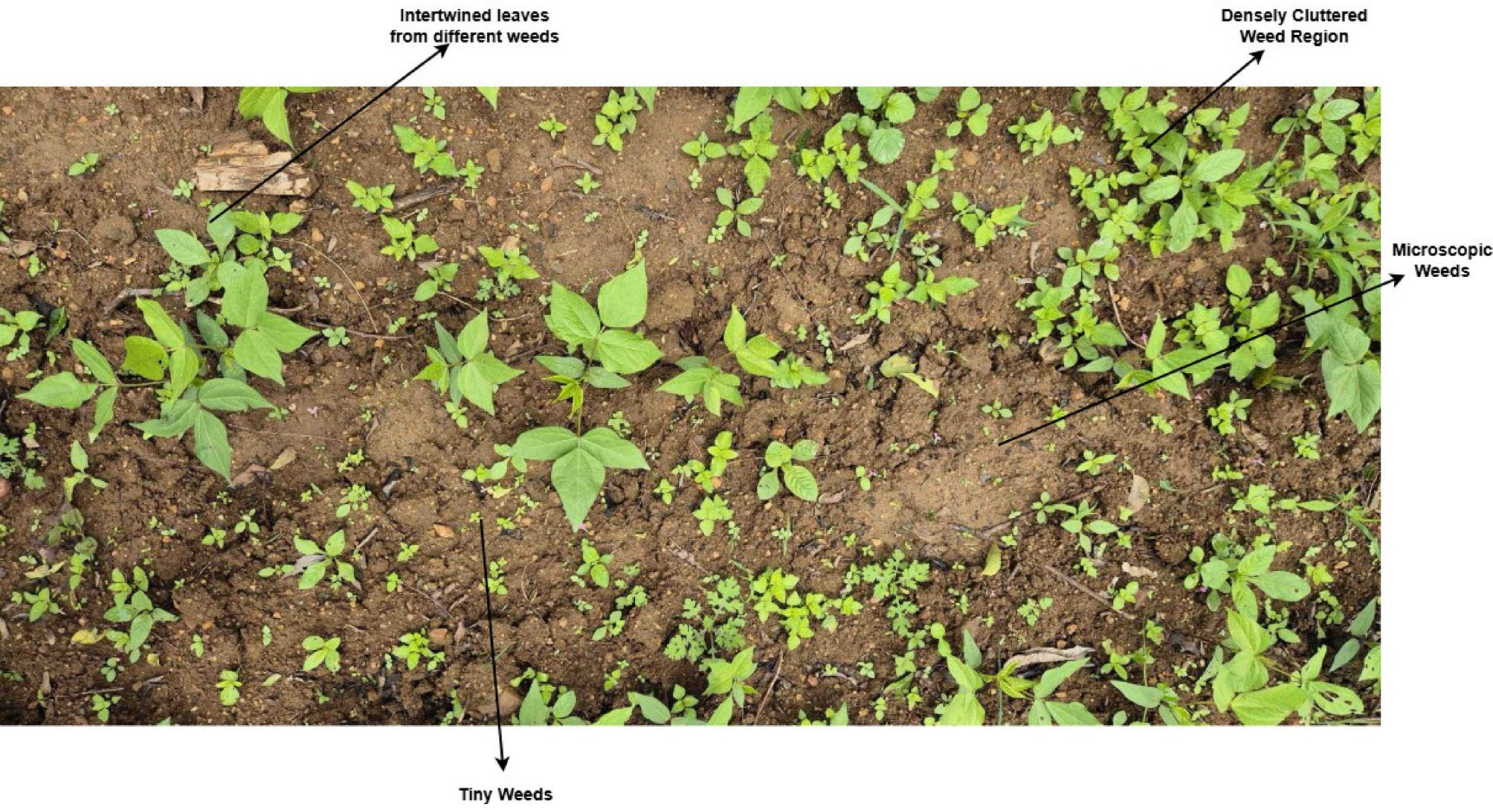
Blackgram field with high weed density and densely packed crop-weed instances making the precise bounding boxes difficult to annotate.

**Fig. 2:**
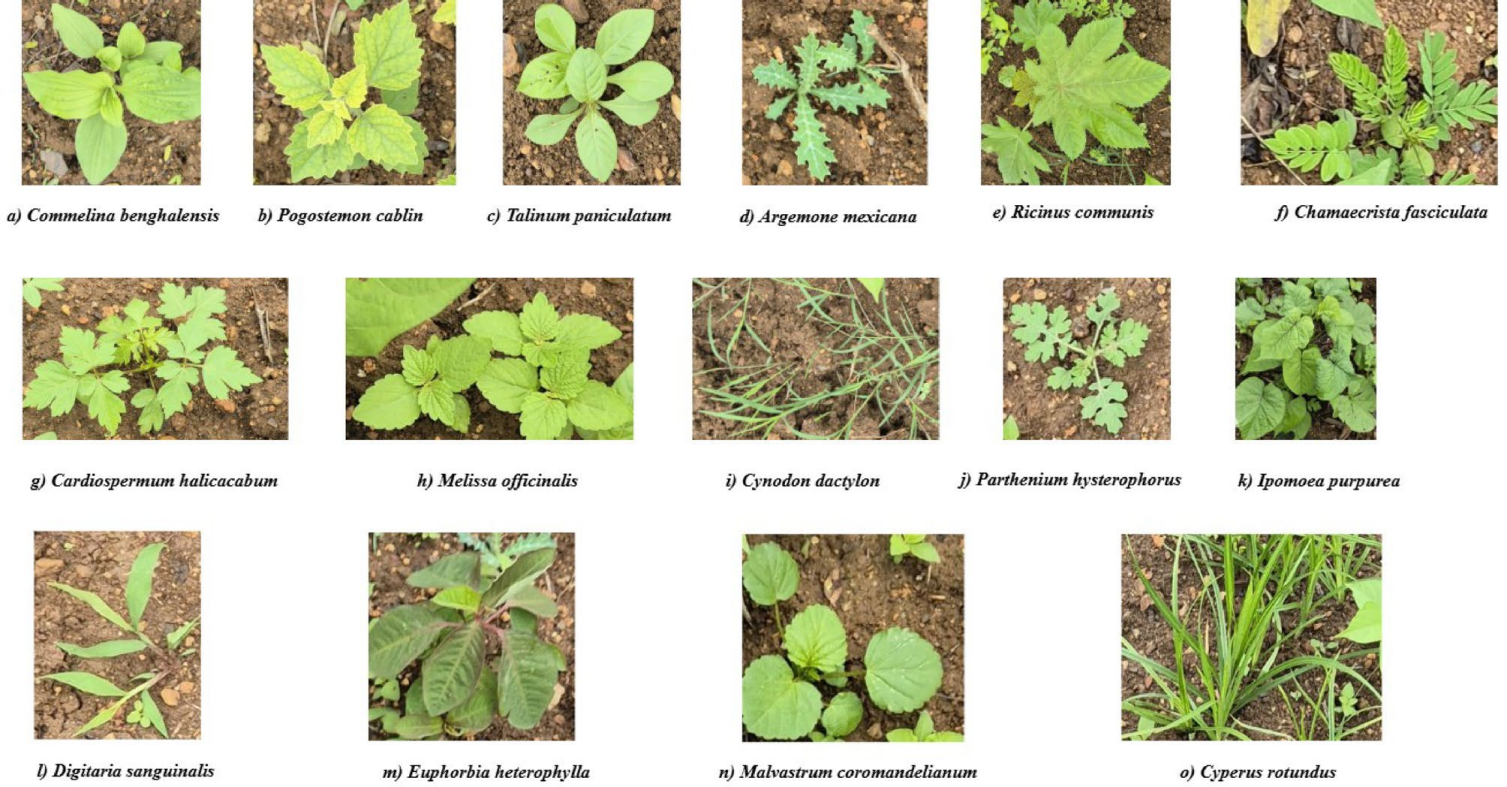
Examples of the different categories of weeds within the dataset, demonstrating the morphology variation in broadleaved and grassy weeds.

Figure 1 depicts the sample blackgram field image from the dataset showing densely cluttered and entangle crop-weed instances. Figure 2 includes the examples of various weed types present in the dataset emphasizing on the morphological diversity.

The proposed model addresses the challenge of detecting multi-stage plant instances in field images, focusing on maturity heterogeneity rather than explicit growth-stage labels. Distinguishes between seedlings of weeds morphologically close to crops and fully mature crops within the same frame, to represent what occurs in an actual field, where weeds appear throughout the cropping stage. Proves to be robust in terms of growth stage diversity and its generalization capabilities across various appearances of mature plants without being provided with stages-specific supervised information or temporal data.

### Image annotation

**Fig. 3:**
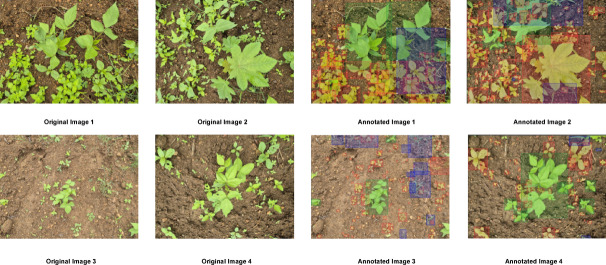
Example of a blackgram field images and its corresponding annotations. The crowded and tangled crop–weed plants illustrate challenges in accurate annotation. Green bounding box represent the blackgram crop, red for broadleaved weed and blue for grassy weeds.

MakeSense.ai is an open source web-based tool that has been used for the purpose of manual object labeling for image annotation. While annotating the image, the bounding boxes were properly delineated for the target classes that included crops and weeds. In order to make the verification process more visible and clear, different colors were selected for the classes such that blue color for grassy weeds, red for broadleaved weeds, while green color for the blackgram crop. The annotated information includes the coordinates of the bounding box along with the class information which were exported in Comma Separated Values (.csv) format to facilitate model training and evaluation. Figure 3 shows the original image vs corresponding annotation.

### Analysis of class distribution

**Fig. 4:**
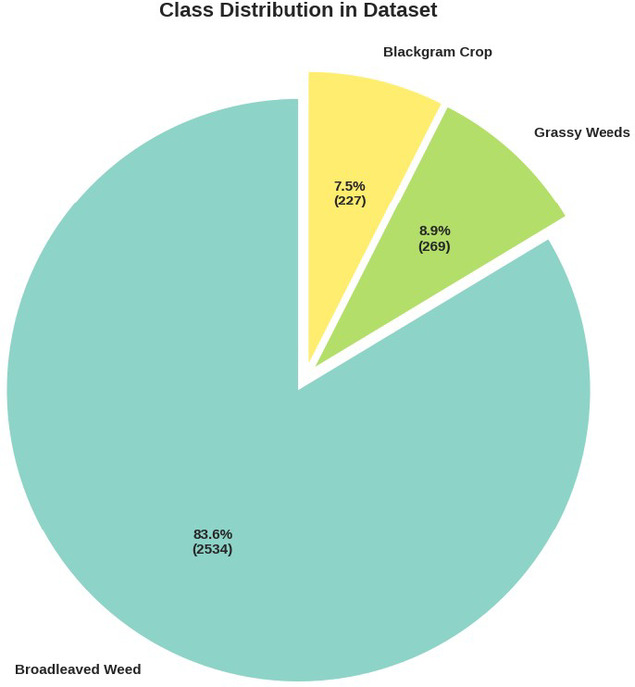
Pie chart showcasing class distribution of crop and weeds.

The dataset includes 3,030 instances of the following three classes: broadleaved weed, grassy weeds, and blackgram crop. Of these, the majority belong to the broadleaved weed class with 2,534 instances. Grassy weeds consist of 269 instances appearing in 74.8% of the total images with an average of 3.02 in an image with a maximum of 11. Blackgram crop with 227 instances in 99.2% of all the images with an average of 1.92 in an image and a highest of 8, suggesting a relatively low density compared to weeds as shown in Fig. 4.

As presented in Table 1, the comprehensive dataset statistics of the blackgram crop weed dataset are shown. Performance-statistic descriptions indicate that the blackgram crop weed dataset presents significant challenges, including the prevalence of tiny weed instances, class imbalance, and a dense presence of crop weed instances.

**Table 1:** Comprehensive dataset statistics of the blackgram crop–weed dataset.

a) Class Distribution	
Class	Count (%)
Broadleaved weed	2534(83.63)
Grassy weeds	269(8.88)
Blackgram Crop	227(7.49)

b) Tiny object statistics (<0.5%)	
Category	Value
Total tiny Objects	2024 (66.80)
Broadleaved weed	1907 (75.26)
Grassy weeds	108 (40.15)
Blackgram crop	9 (3.96)

c) Object density per image	
Metric	Value
Total images	119
Total objects	3030
Mean objects/image	25.46
Median objects/image	21
Std. deviation	15.04
Min–max objects	6–100

d) Detection Difficulty Distribution	
Difficulty	Count(%)
Easy	420 (13.86)
Medium	2478 (81.78)
Hard	132 (4.36)

**Fig. 5:**
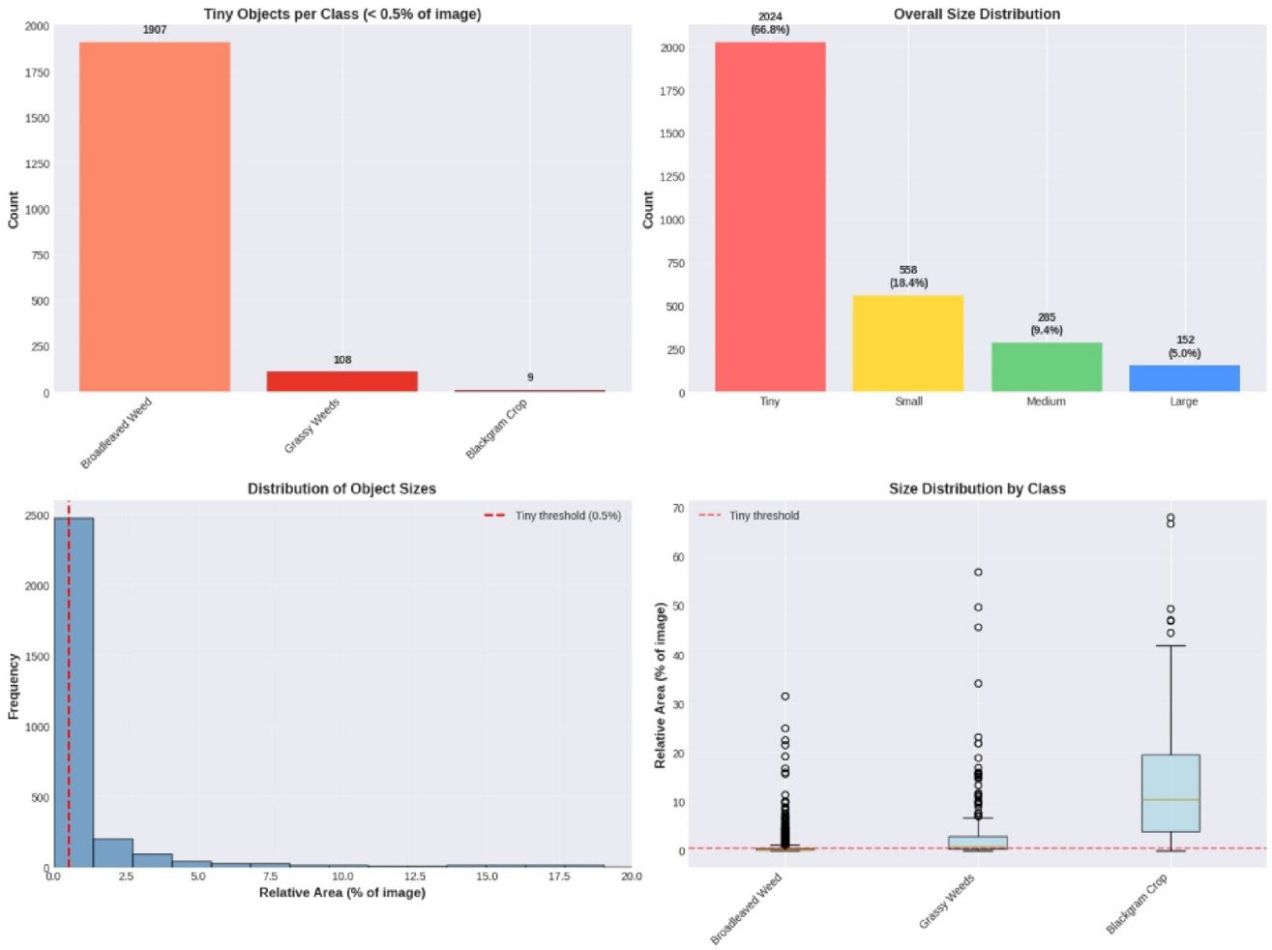
Pictorial representation of dataset statistics.

Table 2 details per class analysis and distribution of crop and weed instances. Figure 5 reveals the pictorial representation of dataset statistics.

**Table 2:** Per-class detailed analysis and distribution of crop and weed instances.

Class Name	Total Instances	# Images Present	% Images Present	Avg per Image (when present)	Avg per Image (overall)	Max in Single Image	% of Total Instances
Broadleaved Weed	2,534	119	100.0%	21.29	21.29	95	83.63%
Grassy Weeds	269	89	74.8%	3.02	2.26	11	8.88%
Blackgram Crop	227	118	99.2%	1.92	1.91	8	7.49%

The proposed dataset is satisfactory because of the size of image collection, diversity, and object annotation density. The dataset contains 119 field images, has 3030 annotations, and on average it contains 25.46 objects per image, while maximum objects in any image is 100 which will improve the training process in comparison with sparse datasets. The proposed dataset provides real world scenario as it contains 83.63% broadleavedweeds,8.88% grass weeds and7.49% blackgram crops which leads to a huge class imbalance and it also comprises of 66.80% tiny objects and 81.78% medium difficulty objects in the scene to test any advanced crop weed detection system.

### Image augmentation

**Fig. 6:**
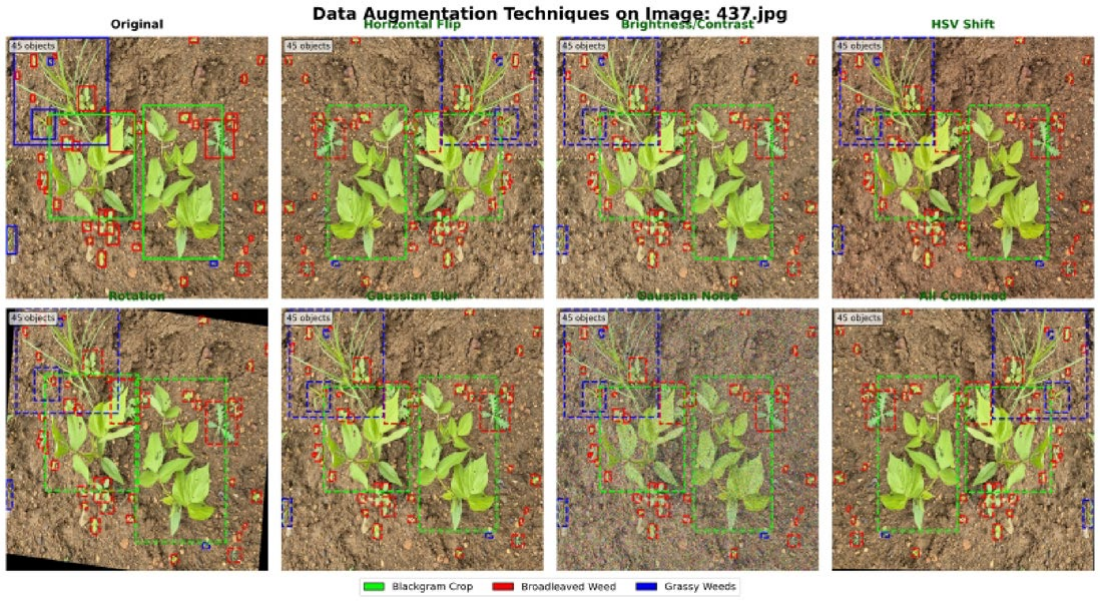
Illustration of the different augmentation strategies applied to a single image.

In order to improve model robustness and generalization capability a data augmentation strategy has been designed using Albumentations library based on agriculture related data and blackgram crop attributes. The applied transformations consist of random horizontal flip (60%), changes in brightness and contrast (40%), change in HSV value (30%), rotation of the image by some random angles (40%) and blur/noise addition to the image (20%). Images were rescaled to 384 × 384 pixels and normalized using ImageNet statistics with pre-trained ResNet-50 architecture. A precise handling of bounding box data was performed to avoid tagging mistakes while applying the augmentation, setting a threshold value to 0.2 on visibility. Applying data augmentation uniformly among images helped in learning diverse features and improve robustness with a focus on learning generic features instead of rebalancing classes. Figure 6 shows the variations in augmentation that were applied to a single image.

### Proposed model

This section introduces the SA-ANMS Faster R-CNN, a proposed framework for effective weed detection in blackgram fields. It enhances the standard Faster R-CNN by integrating a spatial attention mechanism to improve feature discrimination and an adaptive non-maximum suppression strategy to tackle class imbalance and dense object situations.

Table 3 outlines training parameters and hardware configuration.

**Table 3:** Training, detection parameters and hardware configuration.

Parameter	Value
Backbone	ResNet-50 + FPN
Image size	384 × 384
Batch size	6
Epochs	60
Train Test Ratio	80:20
Optimizer	AdamW
Learning rate	0.0005
Weight decay	0.0001
Scheduler	Cosine Annealing
Hardware configuration	
Processor	12th gen intel core i7 (64-bit)
RAM	32 GB
GPU	12 GB Graphics Card
Operating System	Windows 11

#### Baseline Faster R-CNN

The baseline detection framework is based on Faster R-CNN with a ResNet-50 backbone integrated with a Feature Pyramid Network (FPN). Faster R-CNN is a two-stage object detection model composed of the following components:

- Backbone Network: A ResNet-50 Convolutional Neural Network extracts hierarchical feature representations from the input image. The Feature Pyramid Network enhances multi-scale feature learning by combining low-level spatial details with high level semantic information.
- Region Proposal Network (RPN): The RPN generates candidate object proposals by sliding anchors of multiple scales and aspect ratios across the feature maps. Each anchor is assigned an objectness score and bounding box regression offsets.
- Region of Interest (RoI) Head: RoI Align extracts fixed-size feature maps from the proposals, which are subsequently used for object classification and bounding box refinement.

Although Faster R-CNN demonstrates strong performance in generic object detection tasks, its effectiveness is limited in agricultural field conditions due to background clutter, overlapping crop–weed structures, and severe occlusions.

#### Spatial attention module

Unlike existing Faster R-CNN enhancements that apply channel attention (SE-Net, CBAM) after feature extraction as a post-hoc reweighting step, Spatial Attention Module (SAM) is inserted before ResNet Layer4, directly conditioning the backbone’s deepest convolutional stage on spatially discriminative cues. This placement ensures that the network suppresses background clutter—soil texture, crop residue, overlapping canopies—during feature learning rather than after, which is critical in blackgram fields where weed and crop textures are visually near-identical.

Given an intermediate feature map $F^{ \in } {\mathrm{R}}^{C \times H \times W}$, spatial attention is computed by aggregating spatial information using average pooling and max pooling across the channel dimension:

1 $$F_{{{\mathrm{avg}}}} = {\mathrm{AvgPool}}\left( F \right), \;F_{{{\mathrm{max}}}} = {\mathrm{MaxPool}}\left( F \right)$$

The pooled feature maps are concatenated and passed through a convolutional layer followed by a sigmoid activation function:

2 $$A_{s} = \sigma ({\mathrm{Conv}}([F_{{{\mathrm{avg}}}} ;F_{{{\mathrm{max}}}} ]))$$

The refined feature map is obtained through element-wise multiplication:

3 $$F^{\prime } = F \odot A_{s}$$

where ⊙ denotes element-wise multiplication. The spatial attention module is embedded before the final residual block of the ResNet-50 backbone, allowing the network to suppress background noise at an early stage of feature extraction.

#### Adaptive non-maximum suppression

To combat this inherent suppression of true positives within dense weed clusters due to the fixed IoU threshold of Standard NMS, an Adaptive Non-Maximum Suppression (ANMS) strategy has been designed. In this approach class-specific IoU thresholds are chosen to minimize unnecessary overlap and combine, to be:

4 $${\mathrm{IoU}}_{{{\mathrm{thr}}}} \in \left\{ {0.{5},0.{4},0.{3}} \right\}$$

with threshold choices for class based on its specific spread characteristics. Spatially spread out classes (e.g., crops) will have a higher threshold than that for a class with more confined/dense spread (e.g., weeds). In this way, the proposed ANMS can keep duplicate detections in weed clusters but will still reject overlap between two adjacent individual plants (e.g. Crops). These class-specific thresholds have been based upon spatial/morphological information gained from a systematic examination of the data as part of the preliminary work on this project.

Crop instances (threshold = 0.5): Crop plants in Blackgram fields are sown with defined inter-plant distance in rows, and thus the associated bounding boxes of individual crops do not overlap naturally due to spatial separation. It is expected that the majority of overlapping detections will come from false positives (which will need to be discarded by the process) rather than true positives (which will simply have their bounding boxes slightly shifted from the ground truth) as is the case with Standard NMS, which would cause loss of nearby true positive detections. Thus, standard threshold value should be fine as in case of object detection task^10^.

Grassy weeds (threshold = 0.4): Grassy weeds tend to be spread opportunistically between crops rows. They form medium dense spatial clusters where their boundary sometimes becomes blurred by neighbors; thus the threshold should be relaxed slightly to avoid eliminating nearby similar bounding boxes of weeds. We chose 0.4 as the threshold which ensures the similarity of bounding boxes due to their spatial proximity.

Broadleaf weeds (threshold = 0.3): Broadleaf weeds are typically the densest grown within weeds, with individuals being frequently obscured by others in tight clusters, where individual weed is difficult to distinguish. It was found from examination of the data, that mean pairwise IoU of the ground truth bounding boxes of broadleaf weeds was roughly 0.28–0.35 under the typical spread conditions for these plants. Our threshold was selected to be just under this natural occurring value to ensure that similar adjacent boxes (which were individually defined as Broadleaf weeds) will be kept in detection.

This ANMS mechanism has been employed only during training time and during test time of evaluation phase, thereby making the training stable whereas improve the recall and precision at time of detection.

#### Overall framework and model contributions

**Fig. 7:**
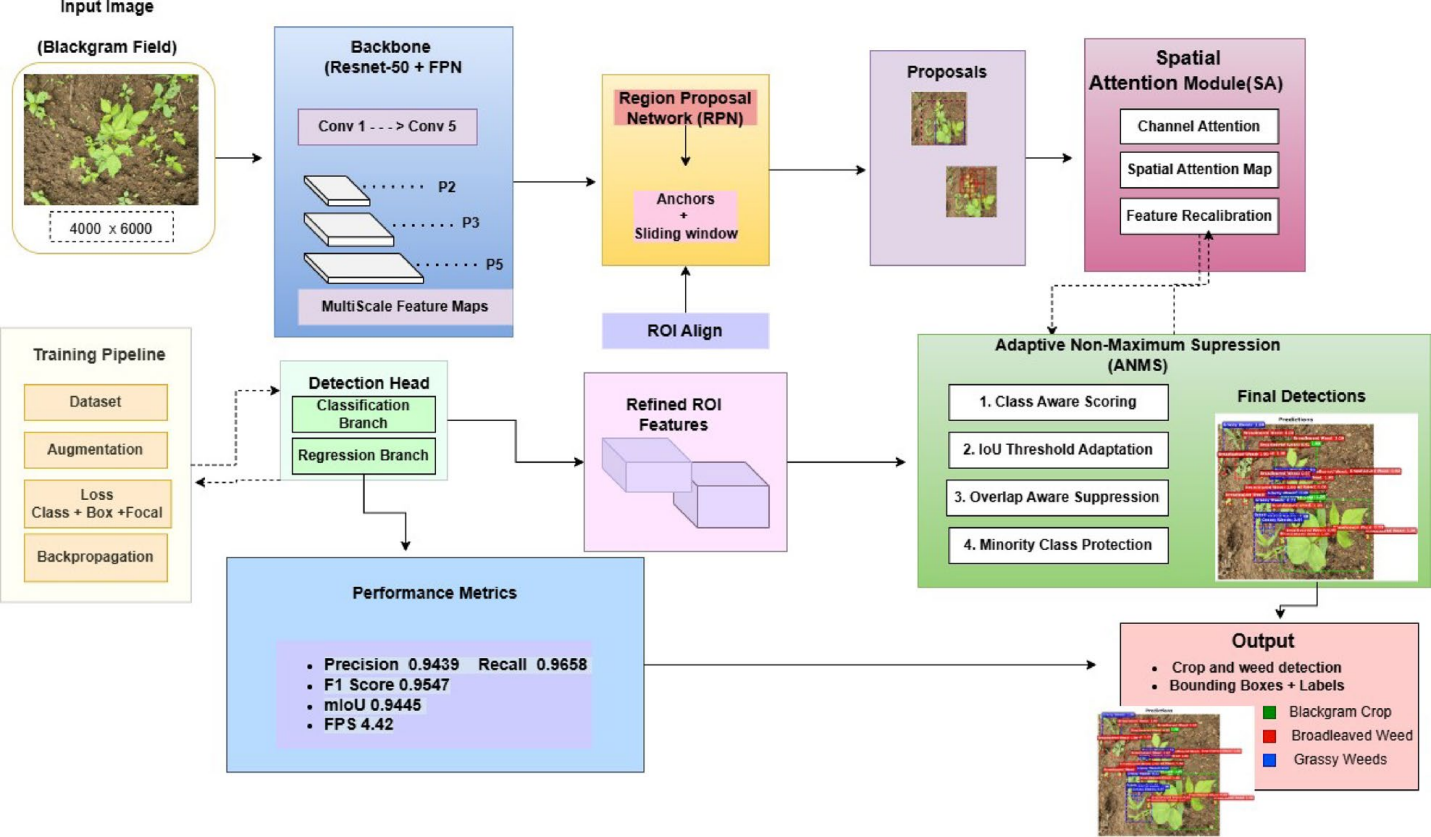
Enhanced Faster R-CNN for crop and weed detection in blackgram fields.

The whole proposed architecture combines spatial attention and adaptive post-processing with Faster R-CNN for improving the performance of crop-weed detection as shown in Fig. 7. The general flow chart of the proposed model can be seen as follow:

1. Input image passes through a Spatial Attention based ResNet-50 with FPN feature extractor to highlight discriminative region.
2. A RPN network predicts potential object proposals corresponding to candidate crops and weeds.
3. For each RoI, feature is extracted and processed by the Faster R-CNN detection head and the category information of RoI canbe predicted along with fine-tuned localization of RoI.
4. A proposed adaptive NMS algorithm using class-specific IoU threshold for detecting dense weed instances.

The SA-ANMS algorithm combining with spatial attention and adaptive post-processing can effectively improve feature discrimination and reduce redundancy detections to enhanceweed detection performance in adverse agricultural environments such as occlusion, clutters, and imbalance of classes.

The SA-ANMS is a novel extension to the existing improvements over Faster R-CNN from three perspectives: First, it employs a Spatial Attention Module prior to ResNet Layer 4 to adaptively condition feature learning according to spatial information, which is a vital factor for distinguishing visually similar textures of weeds and crops. Second, it employs a class-specific Adaptive NMS approach that exploits the characteristic weed morphology to adaptively determine IoU thresholds for weed instances-this approach is novel as none of previous models have addressed it. Last, an ablation experiment of all possible 32 configuration combinations proves that SA + ANMS (F1:0.9547) outperforms all the full five-modules architecture (F1:0.9504) which provides strong evidence that less complicated network may provide better performance in the complex agro-scene.

#### Error analysis and diagnostic evaluation of the proposed model

As shown in Table 4, extensive class-wise error analysis proves that most false positives for all classes are due to background false positives, and in broadleaved weed case, up to 110 out of 130 false positives were from background. This suggest the textures in soil and dense vegetation closely resemble those of weeds. A high amount of false negatives also belongs to small sized objects, (73 out of 84 broadleaved weeds), showing the problem in detecting small object. Double detection was entirely ruled out across all classes, which shows the ability of ANMS mechanism. Mean IoUs for true positives are more than 0.94, and this is a validation of good localization precision.

**Table 4:** Extended class-wise detection performance and error subtype analysis.

Class	TP	FP	FN	Prec	Rec	F1	Mean Iou	Mean Score	FP Cross class	FP Duplicate	FP Low confidence	FP Background	FP No GT	FN Small	FN Med	FN Large	FN Occl
Blackgram Crop	216	21	8	0.9114	0.9643	0.9371	0.9518	0.9962	5	0	0	16	0	2	4	2	0
Broadleaved Weed	2440	130	84	0.9494	0.9667	0.9580	0.9469	0.9971	8	0	12	110	0	73	9	2	1
Grassy Weeds	252	24	14	0.9130	0.9474	0.9299	0.9602	0.9976	10	0	4	10	0	9	5	0	0

The slight drop in F1-score was mainly due to background related false positives, in broadleaved weed cases it’s due to soil texture that are very close to weed structure, and dense vegetation. In other class cases, some amount of false negatives are from small sized weed objects. The above points contribute to the precision and recall trade off, and resulted in the minor fluctuations of class-wise F1.

The total number of cases summed from the confusion matrix will slightly vary from the total number of the datasets since low confidence detections have been filtered and the evaluation involve sIoU-based matching. Such discrepancies are not uncommon in object detection frameworks.

**Fig. 8:**
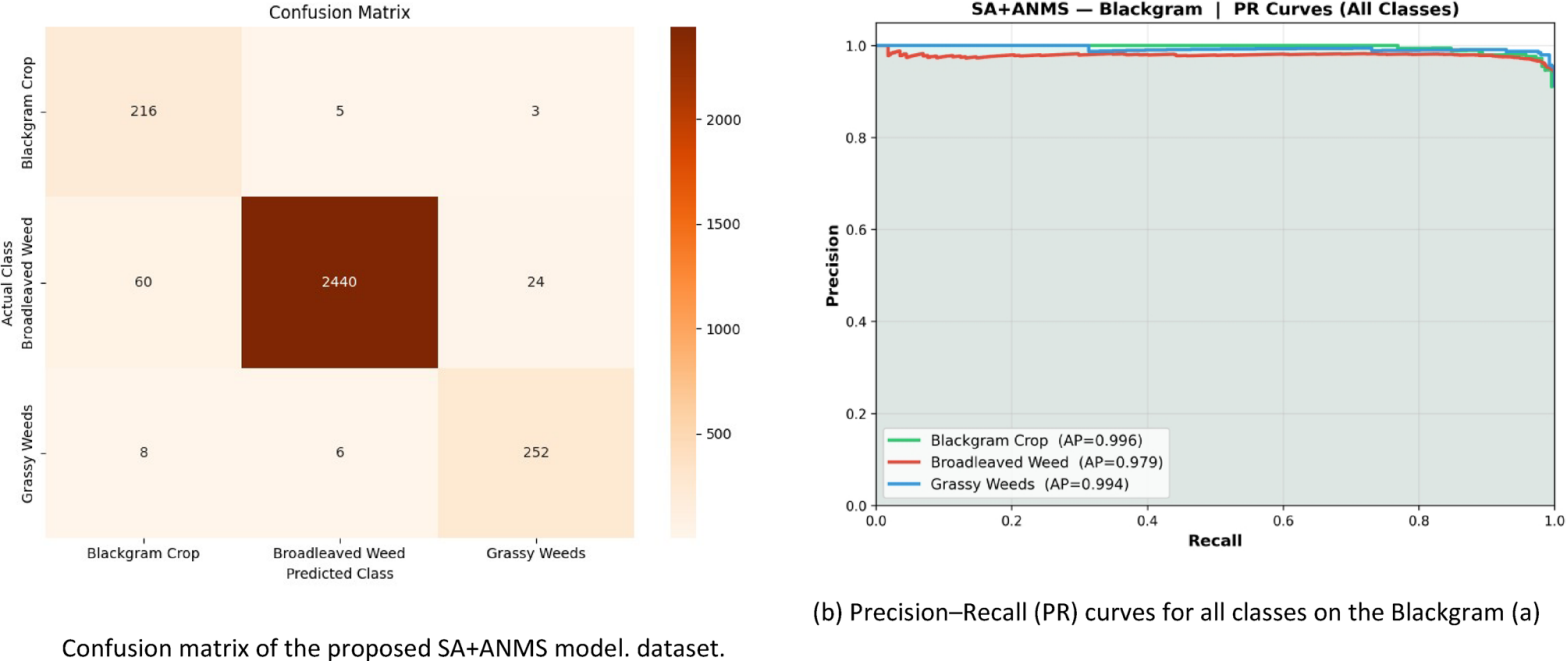
Performance evaluation of the proposed SA + ANMS model on the Blackgram dataset: (**a**) confusion matrix and (**b**) Precision–Recall curves demonstrating strong detection performance across all classes.

The Precision–Recall (PR) curves for the proposed SA + ANMS model demonstrate consistently high detection performance across all classes in the blackgram dataset as depicted in the Fig. 8. The model achieves strong class-wise average precision values of 0.996 for blackgram Crop, 0.979 for broadleaved Weed, and 0.994 for grassy Weeds, indicating robust feature learning and effective object localization. A slight reduction in precision for the broadleaved weed class is observed at higher recall levels, likely due to dense growth patterns and overlapping plant structures. Overall, the PR curves confirm the model’s ability to maintain high precision across increasing recall, validating its effectiveness in multi-class weed detection scenarios.

**Fig. 9:**
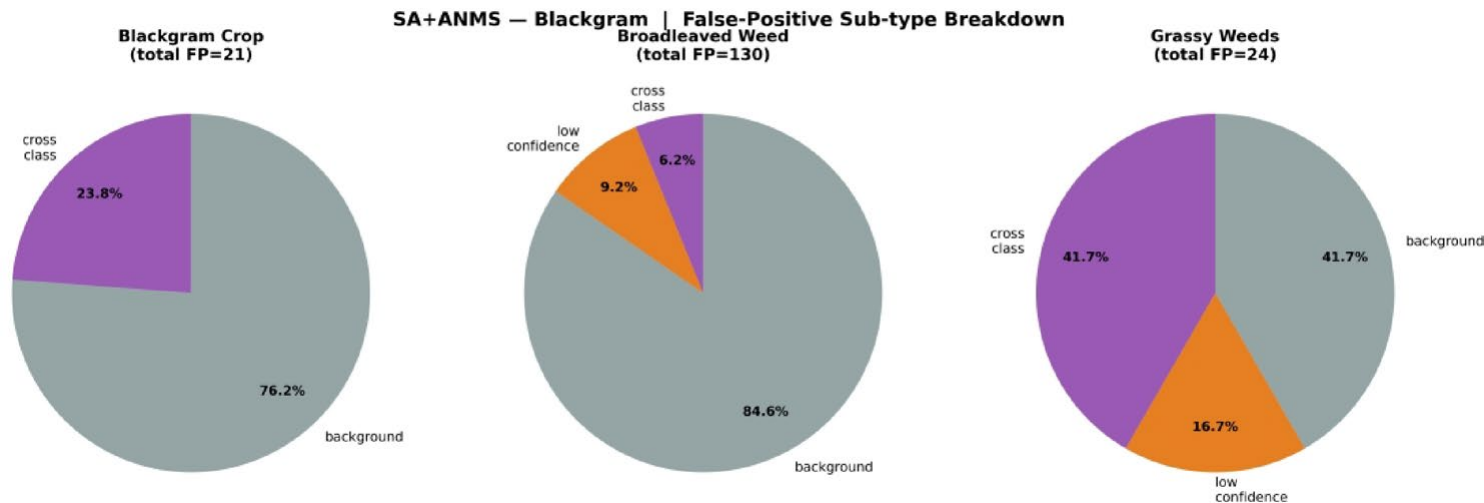
False Positive Breakdown.

The false-positive subtype analysis shown in Fig. 9 reveals that most erroneous detections across all classes are associated with background confusion, particularly for Broadleaved Weeds (84.6%) and Blackgram Crop (76.2%), indicating that visually similar background textures remain a primary source of detection error. Cross-class confusion contributes moderately to misclassification in Grassy Weeds (41.7%) and Blackgram Crop (23.8%), suggesting partial feature similarity between crop and weed structures. Additionally, low-confidence detections are more evident in Grassy Weeds (16.7%) and Broadleaved Weeds (9.2%), reflecting the challenges associated with detecting smaller or densely clustered weed instances. Overall, the analysis highlights that background similarity and inter-class structural overlap are the dominant factors contributing to residual false positives in the proposed SA + ANMS model.

#### Qualitative comparison of ground truth and predictions

**Fig. 10:**
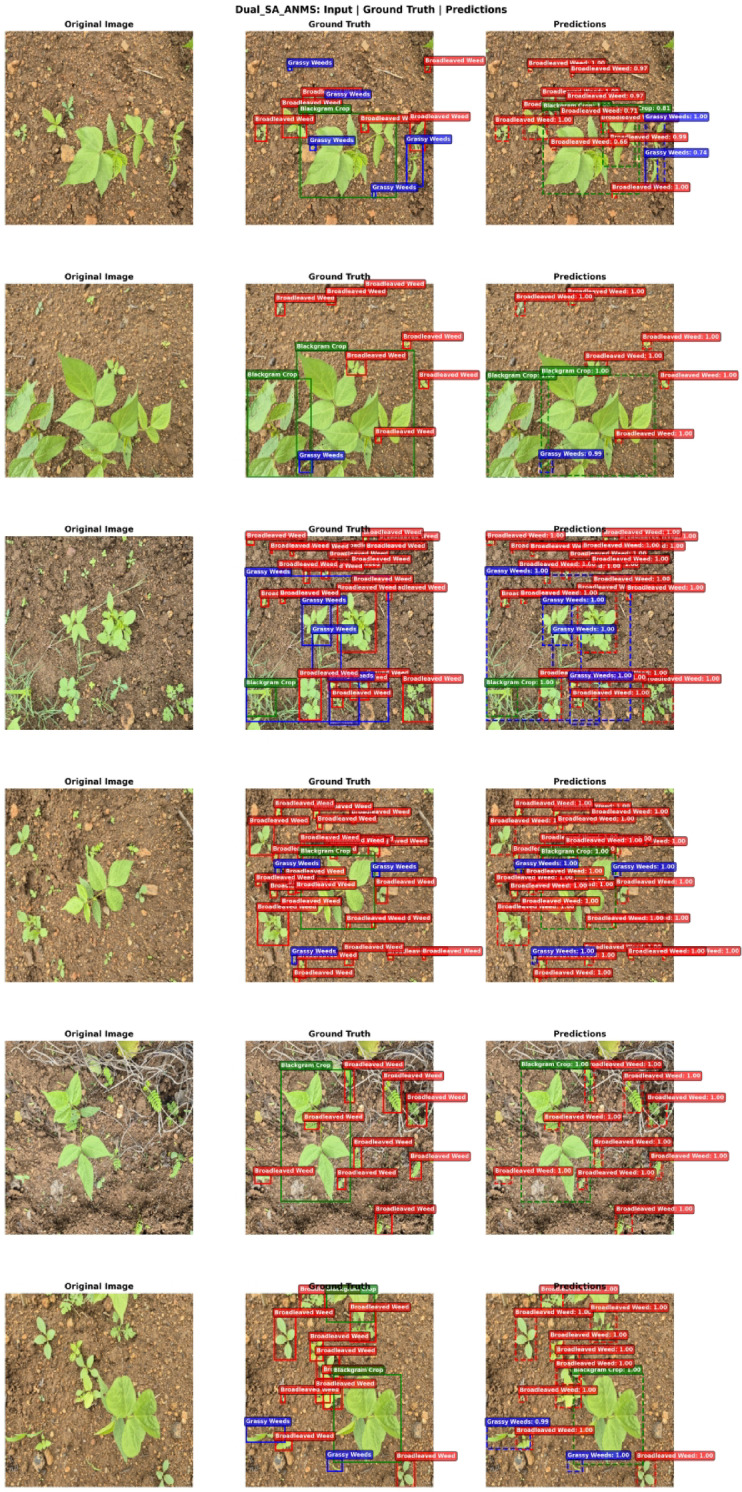
Visualization of input image, ground truth and model output(prediction) of best performing configuration.

Figure 10 depicts the qualitative results of proposed model, where the input image, ground truth, and predicted output are visually analyzed. As shown in the visualization, it is clear that the model is able to effectively represent the weeds despite the severe level of occlusion. Slight deviations in boundaries occur in areas of packed vegetation, but overall, the consistency in spatial representation of the ground truth justifies the robustness of the best configuration.

**Fig. 11:**
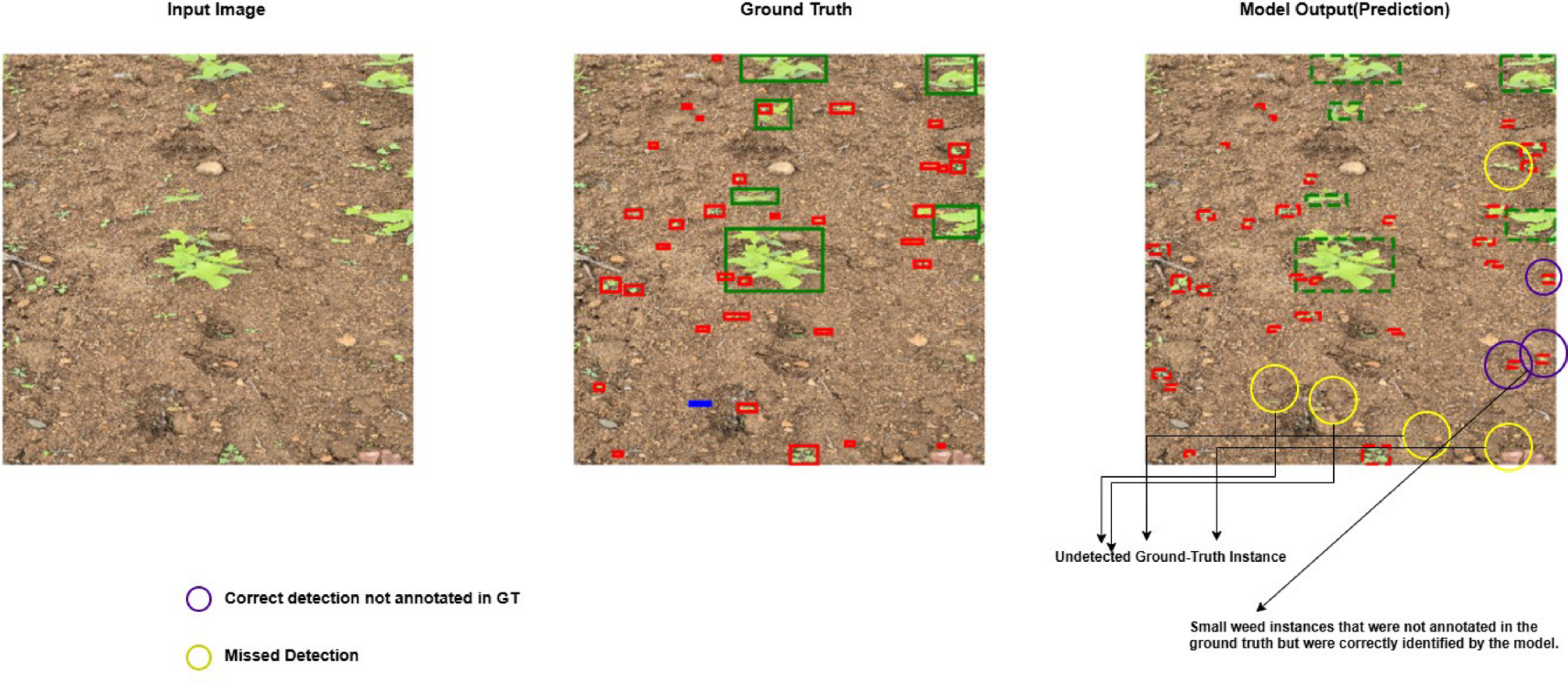
Error analysis of model output.

Figure 11 shows the error analysis of the model’s prediction. It is indicated by the yellow circle marks that the model missed certain detections, meaning that the model was not successful in the detection of the objects as indicated in the ground truth images.

On the other hand, the purple color indicates the detection of objects by the model. Although the model was successful in the detection of these objects, their annotation was not done due to their extremely small size.

## Model generalization on groundnut dataset

To evaluate the generalizability of the proposed SA + ANMS configuration beyond blackgram fields, the model was additionally trained and tested on a groundnut dataset consisting of 273 unique field images collected from Udupi, Karnataka. The model achieved strong performance with a Precision of 0.9288, Recall of 0.9274, F1-score of 0.9281, Accuracy of 0.8659, and mIoU of 0.9401 across groundnut crops, broadleaved weeds, and grassy weeds. These results demonstrate that the proposed configuration can adapt to different crop morphologies and weed types, indicating promising cross-crop applicability.

**Fig. 12:**
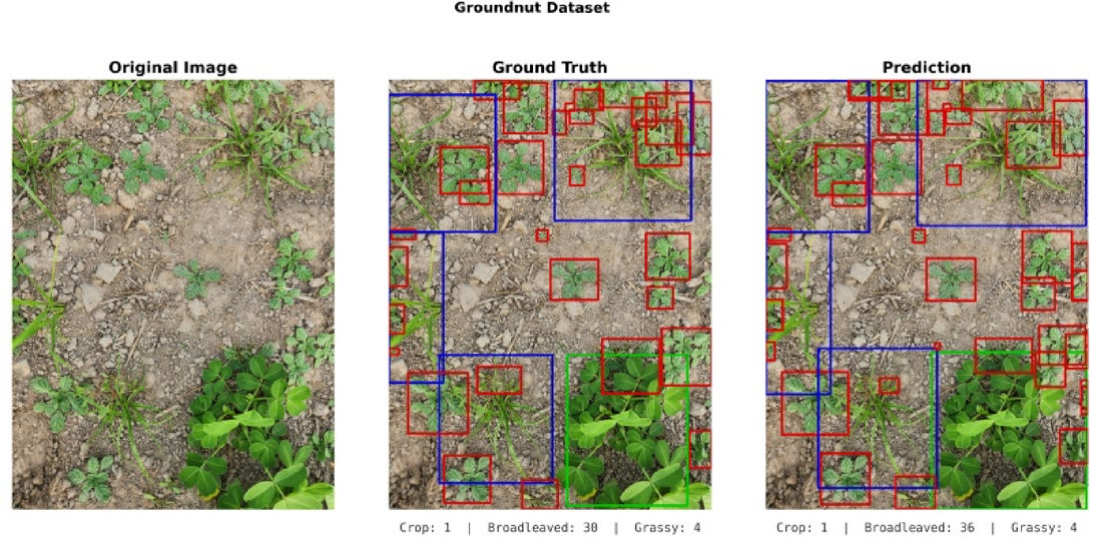
Visualisation of input image, groundtruth and predcition on groundnut dataset.

Tables 5 and 6 reports the overall and per-class performance of proposed model on groundnut dataset.  Fig. 12 illustrates the visualisation of the input image and prediction on the groundnut dataset).

**Table 5:** Overall performance of the proposed SA + ANMS model on groundnut dataset.

Metric	Value
Precision	0.9288
Recall	0.9274
F1-score	0.9281
Accuracy	0.8659
mIoU	0.9401
FPS	4.62

**Table 6:** Per-class performance of SA + ANMS model on groundnut dataset.

Class	Precision	Recall	F1-score	IoU
Groundnut crop	0.9277	0.9434	0.9355	0.9415
Broadleaved weed	0.9388	0.9300	0.9344	0.9422
Grassy weeds	0.8548	0.8691	0.8619	0.9195

## Comparison with other object detection models

Table 7 presents the overall and class-wise performance of different object detection models on the blackgram crop–weed dataset.

**Table 7:** Overall and class-wise performance comparison of object detection models on the blackgram crop–weed dataset.

Model	RetinaNet	EfficientDet	YOLOv5s faster R-CNN		Proposed SA ANMSModel
Overall performance					
Precision	0.1352	0.4686	0.5495	0.9411	0.9439
Recall	0.8469	0.3392	0.6399	0.9695	0.9658
F1-score	0.2332	0.3935	0.5913	0.9526	0.9547
Blackgram crop					
Precision	0.0777	0.0000	0.5750	0.9110	0.9110
Recall	0.9048	0.0000	1.0000	0.9600	0.9640
F1-score	0.1421	0.0000	0.7302	0.9350	0.9370
Broadleaved weeds					
Precision	0.1900	0.4724	0.5461	0.9480	0.9490
Recall	0.8494	0.3868	0.6584	0.9670	0.9680
F1-score	0.3106	0.4253	0.5970	0.9580	0.9590
Grassy weeds					
Precision	0.0280	0.3750	0.0000	0.9000	0.9200
Recall	0.7241	0.1500	0.0000	0.9440	0.9470
F1-score	0.0539	0.2143	0.0000	0.9210	0.9330

The study evaluates the performance of one-stage and two-stage object detectors under extreme class imbalance and dense canopy occlusion. The SA-ANMS model achieved the highest F1-score of 0.9547, surpassing the Faster R-CNN baseline’s score of 0.9526, demonstrating superior precision and recall. In contrast, one-stage models like YOLOv5s and EfficientDet showed poor performance, with YOLOv5s achieving an F1-score of 0.5913 and EfficientDet at 0.3935. In particular, detecting blackgram crops was challenging, with high recall but low precision for YOLOv5s, and very low precision for RetinaNet. For broadleaved weeds, SA-ANMS recorded impressive metrics, while the other models struggled due to occlusion issues. Grassy weeds posed significant challenges, with YOLOv5s failing completely and RetinaNet exhibiting extremely low precision. Overall, the results suggest that lightweight detectors may be inadequate for heavily imbalanced and occluded agricultural datasets, highlighting the effectiveness of Spatial Attention and Adaptive NMS in improving detection accuracy, particularly for minority classes.

The performance discrepancies observed in RetinaNet, EfficientDet, and YOLOv5s highlight crucial characteristics of the blackgram dataset, which drive the necessity for the proposed architectural modifications. RetinaNet shows a significant precision-recall imbalance (P = 0.1352, R = 0.8469), attributed to focal loss miscalibration due to class imbalance, particularly with Broadleaved Weed as the prevalent class leading to a precision cost for high recall. This aligns with known sensitivity of focal loss to class prior mismatches in agricultural detection. EfficientDet exhibits total failure on detecting the Blackgram Crop class (P = 0.000, R = 0.000), while managing partial detection of broadleaved Weed (F1 = 0.4253). This indicates class-specific performance issues tied to the focal loss calibration under the dataset’s imbalanced classes, where the dominant class’s training loss overshadows the minority crop class’s learning drastically. For YOLOv5s, its failure on Grassy Weed (P = 0.000, R = 0.000) is linked to the physical features of Grassy Weed, which fall outside the model’s anchor grid coverage due to their thin elongated shapes. The limitations of this model variant further restrict its ability to differentiate between Grassy Weed and background, indicating that these failures are rooted in anchor-morphology mismatch rather than poor training overall. In summary, the observed failure patterns underscore that applying default COCO configurations to current detection architectures is inadequate for the demands of Blackgram weed detection, thereby reinforcing the need for tailored architectural changes.

**Fig. 13:**
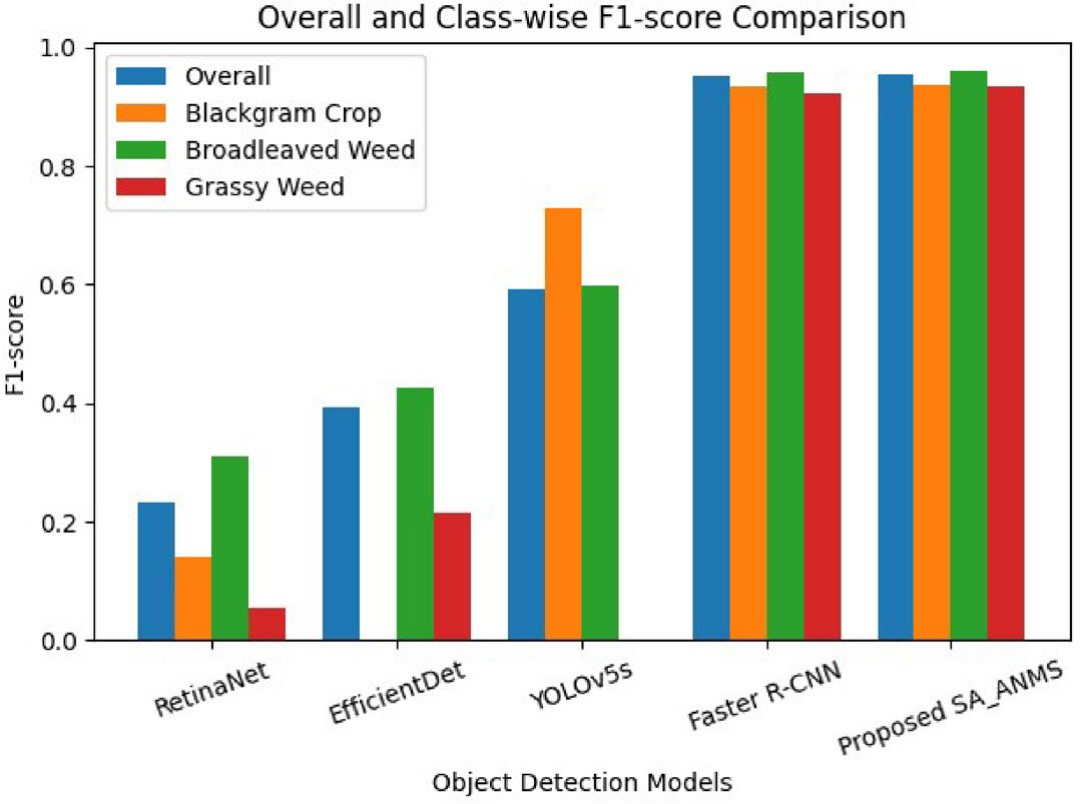
Overall and class wise metrics comparison of various object detection models.

Figure 13 presents the overall and class-wise F1-score comparison across different object detection models. One-stage detectors exhibit severe performance degradation on minority classes, including complete detection failure in certain cases. In contrast, the proposed SA-ANMS Model achieves consistently high performance across all classes, confirming its robustness under dense occlusion and severe class imbalance.

## Ablation study: model and improvements

**Fig. 14:**
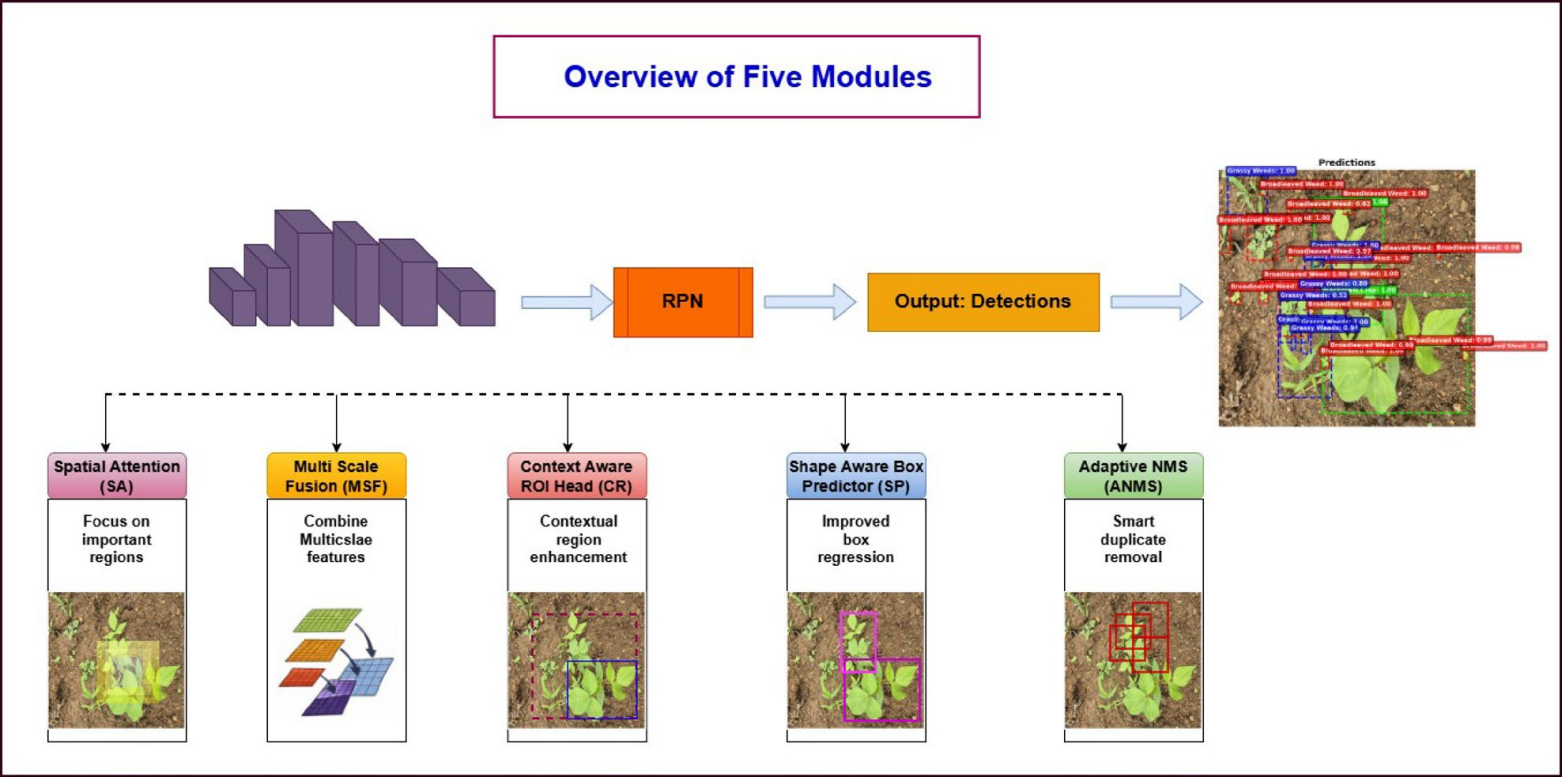
Architectural overview of the five plug-and-play modules integrated into the detection framework: spatial attention, multi-scale fusion, context-aware ROI head, shape-aware box predictor, and adaptive non-maximum suppression.

Figure 14 represents the overview of the five modular components selected for ablation study, including: Spatial Attention, MultiScale Fusion, Context-Aware RoI Head, Shape-Aware Box Predictor, and Adaptive Non-Maximum Suppression.

In order to validate the SA-ANMS model, an experiment on the Faster R-CNN was carried out by training 32 architectural variants from 5 enhancement modules, including Spatial Attention, Multi-Scale Fusion, Context-Aware RoI Head, Shape-Aware Box Predictor, and Adaptive Non-Maximum Suppression. The experiment trained the baseline model ResNet-50 together with all kinds of combinations of above modules for 60 epochs under identical conditions and measured their performance through precision, recall, F1-score, mIoU and FPS. Eventually, SA-ANMS has demonstrated the optimal balance between accuracy and efficiency, and has shown promising application in harsh agricultural conditions of occlusion and class imbalance.

### Individual modules

Table 8 summarizes the proposed model’s description and their functional contributions.

**Table 8:** Detailed description of the proposed modules and their functional contributions.

Module	Description
Spatial attention module (SA)	Produces an attention map which helps the model focus on particular areas of the image, retaining important features like crops and weeds while neglecting non-important background areas like soil and shadows. The attention mechanism is highly suitable for use in images pertaining to agriculture because, in this scenario, the tiny weed might get overshadowed by the surrounding noise. In the case of blackgram fields, where the color distribution of both crops and weeds is almost the same, the technique focuses on important structural points like stem joints and leaf edges
Multi scale fusion (MSF)	This technology boosts the network’s ability to identify objects at various spatial scales by blending global context with local spatial details. It uses a pooling function to gather global context, which is then spread across different spatial locations through convolutional layer. This feature is especially crucial in agricultural settings, where crops and weeds can vary significantly in size, allowing for precise detection and classification of objects, no matter their scale
Context-aware ROI head (CR)	This approach enhances region-level classification by pulling high-dimensional feature representations (1024 channels) from each identified region and evaluating their importance on the fly. It’s particularly useful in agricultural settings where objects that look alike have subtle differences. However, when there’s heavy occlusion, the module might accidentally pick up features from overlapping objects, which can mess with the accuracy of feature reweighting. This work dive deeper into this limitation in the ablation analysis to see how it affects detection accuracy
Shape-aware box predictor (SP)	Introduces an auxiliary prediction branch that estimates geometric properties of detected objects, particularly width–height ratios associated with plant morphology. While the primary prediction head focuses on object classification and bounding box localization, the shape branch learns structural characteristics that distinguish plant types. This allows broadleaf weeds to be identified as wide and rounded structures, narrowleaf crops as moderately compact forms, and grassy weeds as tall and elongated structures. Such morphological awareness reduces classification ambiguity and improves bounding box regression accuracy
Adaptive non maximum suppression (ANMS)	Enhances duplicate detection removal by applying class-specific suppression thresholds rather than a single fixed Intersection-over-Union (IoU) threshold. Unlike conventional NMS, which typically uses a uniform threshold (e.g., 0.5), the proposed ANMS employs adaptive thresholds of 0.5 for crops (strict suppression), 0.4 for grassy weeds (moderate suppression), and 0.3 for broadleaf weeds (lenient suppression). This design specifically addresses challenges associated with densely packed broadleaf weeds, where conventional NMS may incorrectly suppress overlapping detections as duplicates. By adjusting suppression behavior according to class-specific spatial distribution patterns, ANMS improves detection sensitivity in dense vegetation while maintaining localization precision for isolated crop instances

**Fig. 15:**
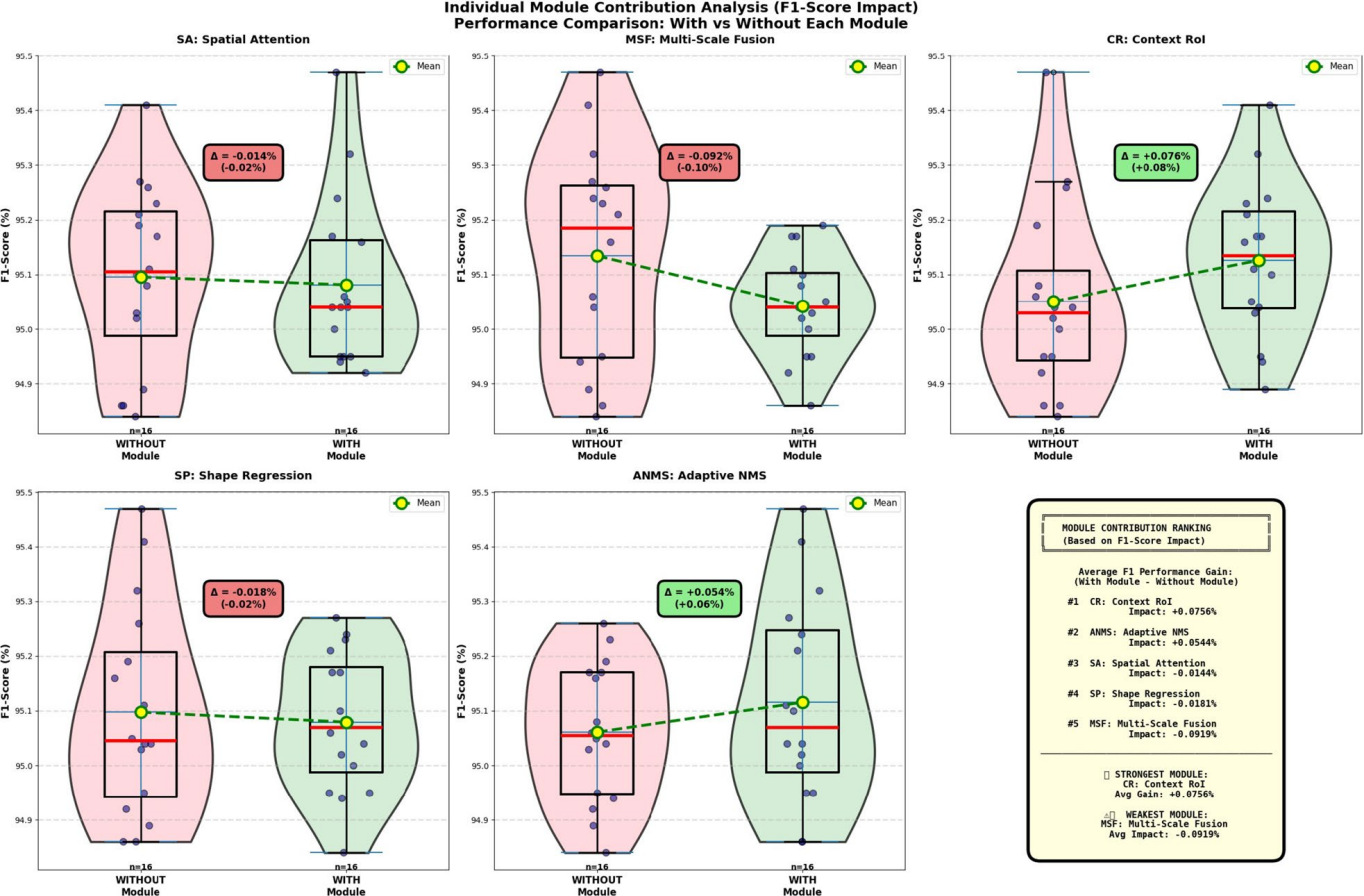
Contribution of individual modules.

Figure 15 reveals the contribution of individual modules.

### Analysis of findings

This section synthesizes insights derived from the comparative evaluation and the systematic ablation-based model selection, with emphasis on detection robustness under severe class imbalance, dense canopy occlusion, and extreme scale variation.

#### Effect of module combinations

This analysis demonstrates that integrating multiple modules yields superior performance compared to using individual modules. Observations indicate enhanced object detection with combined approaches like SA or CR alongside ANMS, improving recall and F1-score, while also refining the definition of boundaries and class distinction. However, leveraging multiple modules may introduce complexity. An ablation study illustrates how the addition or removal of components affects detection performance through their interaction patterns, confirming that optimal results come from well-balanced interactions instead of excessive stacking.

The ablation study on 32 architectural settings shows important findings on the role and interaction of different components. The baseline model Faster R-CNN serves as a solid foundation with evaluation metrics of F1 = 0.9526 and mIoU = 0.9446. The addition of individual components demonstrates incremental improvement, where Spatial Attention improves the discrimination of features, Adaptive NMS refines the predictions of overlaps, and Contextual Representation maintains consistency in contextual reasoning. However, Multi-Scale Features (MSF) provides only moderate benefits and does not consistently improve performance with aggressive combinations.

**Fig. 16:**
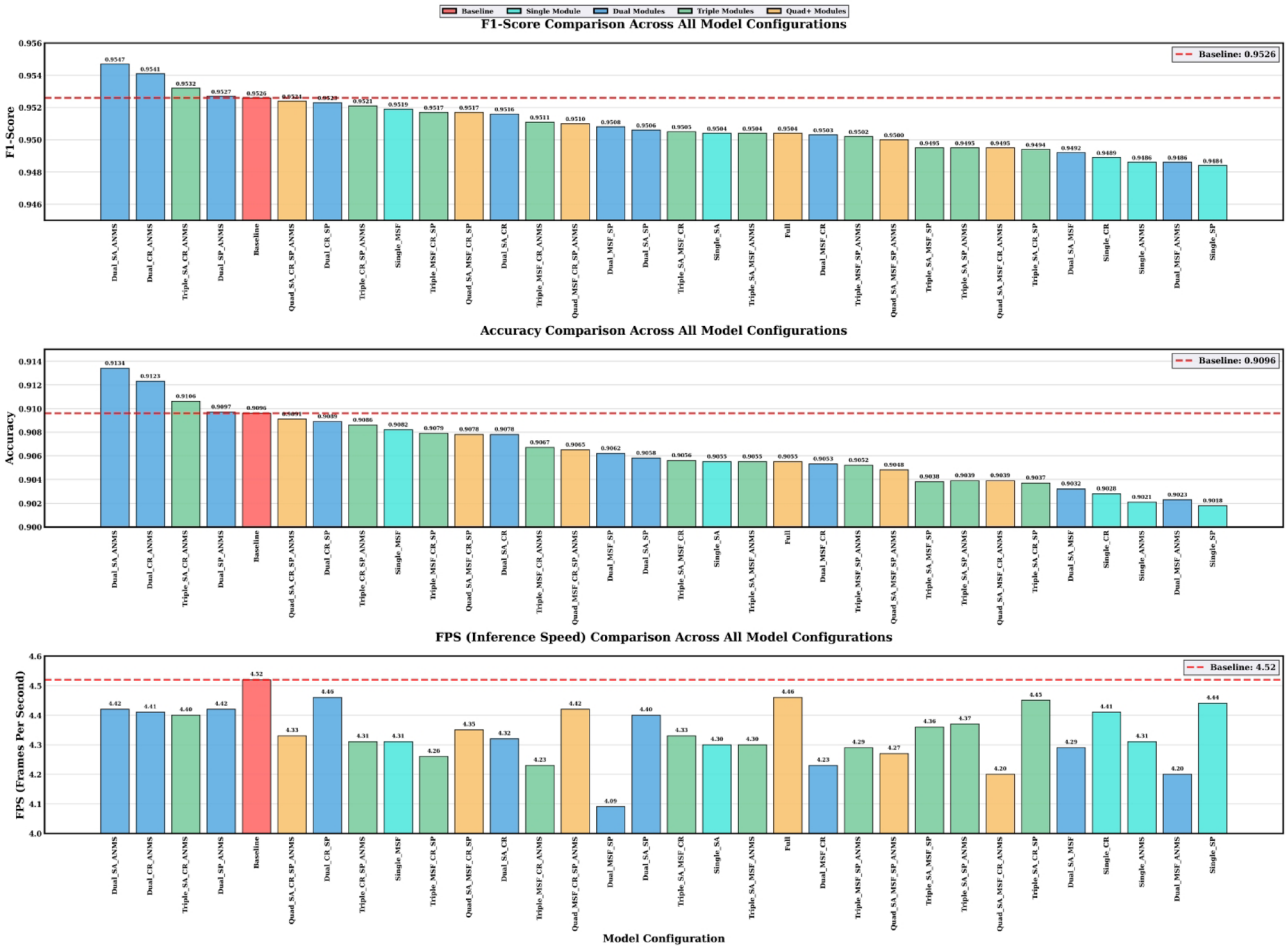
F1-Score, accuracy and FPS comparison across all model configurations.

The top-performing configurations consistently involve Adaptive NMS (ANMS) along with complementary feature-enhancing modules. The Dual SA-ANMS configuration achieved the best detection performance, showing significant F1-score, accuracy, and mIoU values, indicating effective cooperation between spatial attention and ANMS for improved feature learning without increased complexity. Moreover, the results from Dual CR-ANMS and Triple SA-CR-ANMS further support that ANMS enhances boundary representation, avoiding misclassification in overlapping areas of crops and weeds. This indicates strong synergistic effects between attention or contextual modeling and adaptive post-processing.  Fig 16 illustrates the comparison of F1 Score, accuracy and FPS across all model configuration using bar graph.

In contrast, Full and MSF-heavy models exhibit slight performance degradation, indicating that excessive feature fusion may lead to instability and inefficiency. A consistent pattern observed across 32 configurations shows that any model containing MSF underperforms against its MSF-free counterpart, reaffirming systematic interference instead of random variance, primarily due to background signal conflicts and scale imbalance affecting small-instance recall. Conversely, configurations that excessively stack modules, including the complete five-module integration, fail to improve results and may even slightly degrade performance, suggesting challenges related to feature competition and optimization instability.

Overall, the findings highlight that improved performance arises from focused spatial refinement rather than merely combining various modules, with dual and triple combinations being the most effective regarding accuracy, robustness, and computational efficiency.

**Fig. 17:**
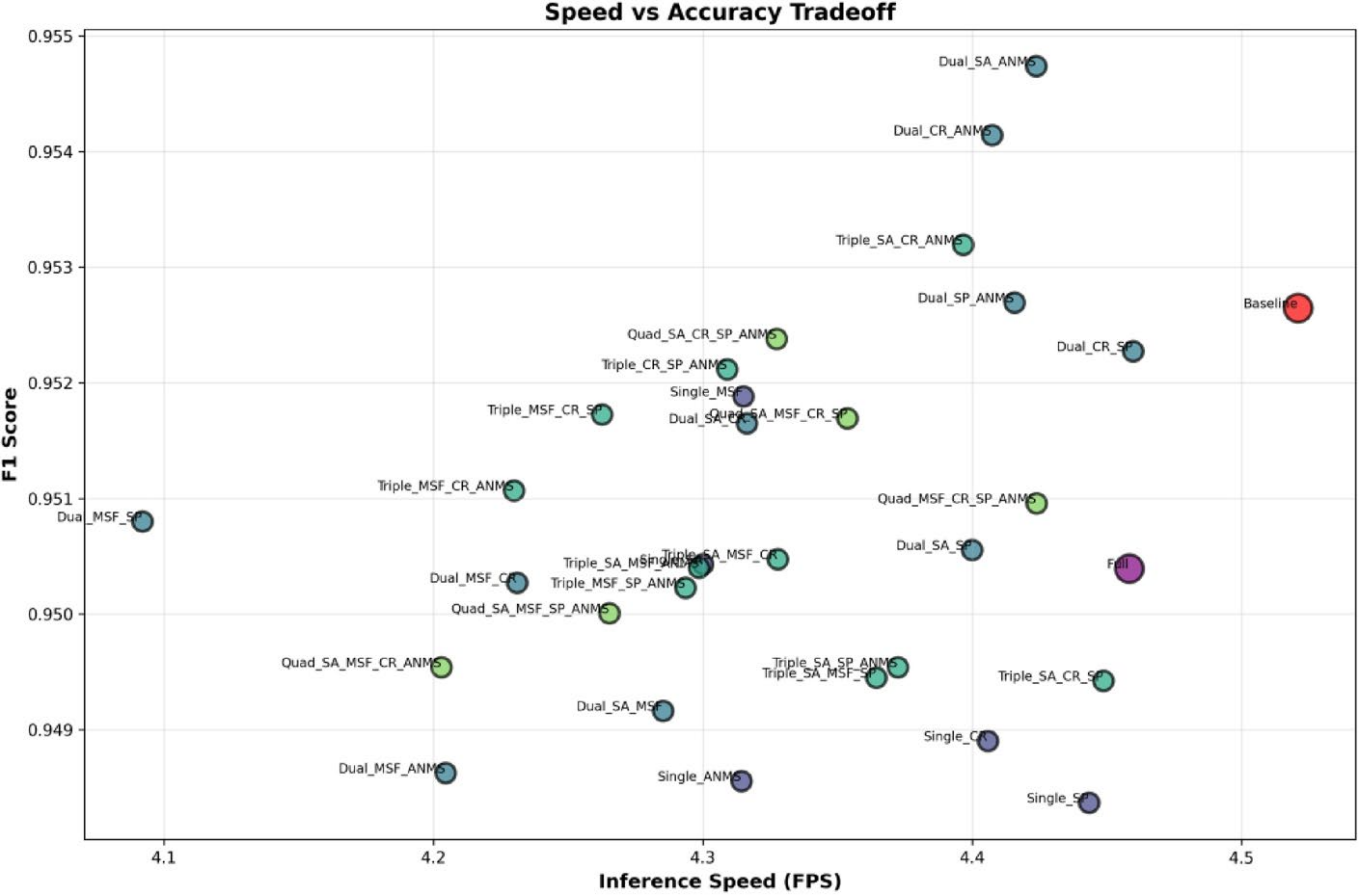
Illustration of the speed vs accuracy tradeoff for all 32 configurations.

Figure 17 illustrates the speed–accuracy trade-off across the 32 evaluated configurations.

#### Mechanistic Interpretation of Module Interactions

- Mechanism Behind Performance Improvement with Spatial Attention (SA) Channel-pooled attention by summing concatenated average and max-pooled maps results in the SA module that precedes ResNet layer 4 to re-weight activations along the spatial axis. These weights effectively suppress background textures and boost discriminant border regions of the Blackgram dataset before features are passed to FPN; as crops and weeds tend to be scale-common and somewhat occluded. These features make a benefit for region proposal that immediately follows, by directly using such cleaned features.
- Role of Adaptive Non-Maximum Suppression (ANMS) as a Key Performance Driver: In the Blackgram detection setting,frequent overlapping of spatially inter-class locations (i.e., common co-occurrence of broadleaved and grassy weeds), standard NMS with one constant threshold value is not suitable. Using different suppression thresholds (0.5 for crop, 0.3 for broadleaved, 0.4 for grassy weed), ANMS helps in reducing the false duplicates in the sparser regions as well as detecting the missed detections in dense regions. Since SA + ANMS benefit is additive, it is without interferences of architecture, due to its post-hoc additive nature that is orthogonal to modifications on backbone or RoI head.
- Analysis of Performance Degradation Associated with Multi-Scale Fusion (MSF): Each level of the FPN is independentlyapplied the global average pooling, nearest neighbor up sampling, and residual add through the Lightweight Multi-Scale Fusion module. The single scalar context extracted from each level is not enough to preserve the heterogeneity in weed distributions over space and the residual is injected into it, disrupting the exact trained inter-level feature balances produced by FPN and causing an information bottleneck. The gradient competition issue exacerbates the situation as well: MSF downstream tries to put back the global context that SA has erased earlier on, resulting in conflicts between the gradients during backpropagation, whereas SA upstream tends to direct the spatial gradients to locations of high prominence.
- Analysis of Standalone Performance Contribution of Context-Aware RoI (CR): By using adaptive average pooling over a 7 × 7 RoI grid to create a global channel-wise weight vector, which is then applied as a multiplicative gate, the Context-Aware RoI Head improves RoI-pooled features. The goal of this procedure is to enhance RoI channels that are pertinent to object appearance while suppressing those that are not. However, because Faster R-CNN’s RoI Align already normalizes proposals, eliminating considerable spatial context, its efficacy in the Blackgram detection situation is constrained by the RoI feature distribution. As a result, the CR module uses little spatial information, which results in a small and uneven standalone contribution during training runs.
- Analysis of Gradient Competition Effects Introduced by Shape-Aware Prediction (SP): By adding a third branch that uses a shared RoI feature vector to forecast aspect ratio and scale parameters for each class, the Shape-Aware Box Predictor improves upon the conventional dual-head predictor. By aligning it with the normal morphological shapes of weeds, this method seeks to improve box regression. However, when compared to baseline configurations, the shared feature representation causes competing gradient pressures during backpropagation, impeding the principal detection aims and producing minimal or negative contributions to F1 scores.
- Interaction Effects Between Context-Aware RoI (CR) and Shape-Aware Prediction (SP): Failure modes for the combinationsof CR and SP yield more complex problems. The gradient competition between the three gradient streams produced by SP does not occur without performance loss if the feature vector for the RoI is first recalibrated by CR before the box head. CR reduces feature redundancy by suppressing channels, so that the gradient competition for SP may occur at negligible cost to performance. Combinations with CR + SP all create a brittle representation that is far too partitioned by SP and compressed by CR to result in high performance.
- Performance Trends Associated with Progressive Module Accumulation: The degradation seen with the introduction of mod-ules on top of SA + ANMS is attributed to cumulative interference, not increased capacity. When applied on top of SA modulated backbone features, the Context RoI (CR) module uses global channel weights to rescale RoI features, which leads to the repetition of transformations and loss of feature diversity for the box predictor. The essential classification and localization objectives in the final layers of the Shape Predictor (SP) do not perform as well because of a conflicting auxiliary prediction branch. The competition among prediction branches illustrates that adding all five modules together leads to performance comparable to that of only a SA module, because interference nearly cancels out the benefit of an increased architecture.

**Fig. 18:**
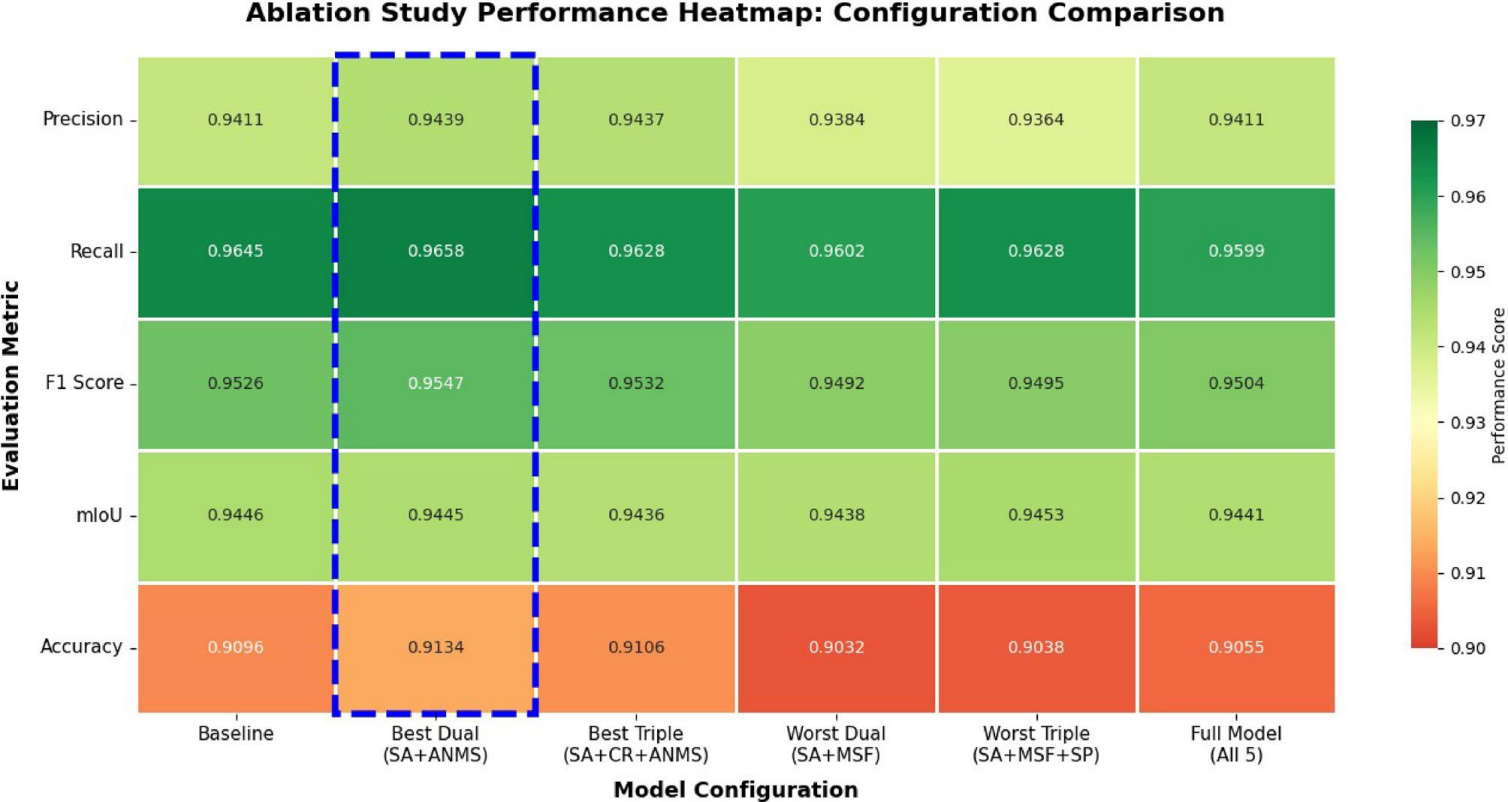
Heatmap visualization comparing ablation results for six selected configurations against five metrics.

The overview of five modules is depicted in Table 9. Figure 16 displays the F1 score, accuracy, and FPS for all configurations, and Fig. 18 shows a heatmap-based analysis of six settings using five metrics for comparison.

**Table 9:** Overview of the five modules and their impact on performance.

Module	Acronym	Purpose	Location in network	Impact on results
Spatial attention	SA	Focus on important regions	After backbone, before RPN	Positive
Multi-scale fusion	MSF	Combine features at different scales	Feature pyramid	Positive
Context-aware RoI head	CR	Enrich region features with context	RoI head	Positive
Shape-aware box predictor	SP	Improve bounding box regression	Box regression head	Synergistic
Adaptive NMS	ANMS	Smart duplicate removal	Post-processing	Synergistic

#### Feature-level interactions underlying multi-module performance degradation

There are several levels of features being interacted across multiple modules and cause degradation. The performance degradation of the multiple-module system stems from a chain of interactions at feature-level that cause an erosion of the detection pipeline integrity, not by a particular module. Three kinds of feature interactions are specified.

- Mechanism 1: FPN Inter-Level Coherence Disruption by MSF
- FPN produces feature maps of different levels(P2-P5) via pretraining and detection training. Each level maintains relation between different scales, which are crucial for object detection. The MSF module breaks this relation, by employing global average pooling and nearest-neighbor upsampling at each level, independently. Consequently, it generates the same context vector for each level and therefore, attenuates spatial contrast. Hence, the calibrated magnitude relation, required for correct scale-consistent scoring of proposals, is broken, giving rise to polluted confidence scores for regions proposals. This causes a reduction in recall in multiple scales.
- Mechanism 2: Upstream–Downstream Gradient Conflict Between SA and MSF
- In Dual SA-MSF, placing SA upstream and MSF downstream leads to conflicting gradient pressures at the interface of backbone-FPN: SA increase the spatial gradient at the foreground, thus creating high magnitude activation at its location; whereas the pooling and residual connection used in MSF attempt to increase the global context which is decreased by SA. This create conflicts at the backpropagation: loss gradient for MSF would want to modify feature where SA wants to suppress. Eventually, performance of Dual SA -MSF is always worse than that of Single SA for each evaluation indicator. This has been described in Table 10.
- Mechanism 3: Representational Bottleneck Accumulation Across Sequential Modules
- All the modules in pipeline are dimensionality reduction of features, SA suppresses spatial locations, CR suppresses channels, MSF equalizes spatial contrast, and SP splits RoI representations. While residual connections in CR and MSF help preserve the original signal, they do not prevent gradient interference. As the number of modules increases, the information content for the box predictor diminishes because each module uses a compressed representation, leading to diminishing returns in transformations. This results in the full five-module model performing similarly to a single-module SA due to the cumulative bottleneck effect on features.

**Table 10:** Performance comparison of all 32 configurations, ranked in descending order of F1-score, with the highest F1-score at the top and the lowest at the bottom.

Configuration	Modules	Precision	Recall	F1	Accuracy	mIoU	FPS
Dual SA ANMS	SA + ANMS	0.9439	0.9658	0.9547	0.9134	0.9445	4.42
Dual CR ANMS	CR + ANMS	0.9421	0.9665	0.9541	0.9123	0.9441	4.41
Triple SA CR ANMS	SA + CR + ANMS	0.9437	0.9628	0.9532	0.9106	0.9436	4.40
Dual SP ANMS	SP + ANMS	0.9434	0.9622	0.9527	0.9097	0.9450	4.42
Baseline	None	0.9411	0.9645	0.9526	0.9096	0.9446	4.52
Quad SA CR SP ANMS	SA + CR + SP + ANMS	0.9396	0.9655	0.9524	0.9091	0.9434	4.33
Dual CR SP	CR + SP	0.9416	0.9632	0.9523	0.9089	0.9450	4.46
Triple CR SP ANMS	CR + SP + ANMS	0.9413	0.9632	0.9521	0.9086	0.9437	4.31
Single MSF	MSF	0.9393	0.9648	0.9519	0.9082	0.9445	4.31
Triple MSF CR SP	MSF + CR + SP	0.9390	0.9648	0.9517	0.9079	0.9423	4.26
Quad SA MSF CR SP	SA + MSF + CR + SP	0.9427	0.9608	0.9517	0.9078	0.9448	4.35
Dual SA CR	SA + CR	0.9404	0.9632	0.9516	0.9078	0.9443	4.32
Triple MSF CR ANMS	MSF + CR + ANMS	0.9383	0.9642	0.9511	0.9067	0.9429	4.23
Quad MSF CR SP ANMS	MSF + CR + SP + ANMS	0.9403	0.9618	0.9510	0.9065	0.9446	4.42
Dual MSF SP	MSF + SP	0.9400	0.9618	0.9508	0.9062	0.9448	4.09
Dual SA SP	SA + SP	0.9383	0.9632	0.9506	0.9058	0.9451	4.40
Triple SA MSF CR	SA + MSF + CR	0.9397	0.9615	0.9505	0.9056	0.9438	4.33
Single SA	SA	0.9374	0.9638	0.9504	0.9055	0.9446	4.30
Triple SA MSF ANMS	SA + MSF + ANMS	0.9380	0.9632	0.9504	0.9055	0.9453	4.30
Full	SA + MSF + CR + SP + ANMS	0.9411	0.9599	0.9504	0.9055	0.9441	4.46
Dual MSF CR	MSF + CR	0.9402	0.9605	0.9503	0.9053	0.9448	4.23
Triple MSF SP ANMS	MSF + SP + ANMS	0.9379	0.9628	0.9502	0.9052	0.9448	4.29
Quad SA MSF SP ANMS	SA + MSF + SP + ANMS	0.9388	0.9615	0.9500	0.9048	0.9434	4.27
Triple SA MSF SP	SA + MSF + SP	0.9364	0.9628	0.9495	0.9038	0.9453	4.36
Triple SA SP ANMS	SA + SP + ANMS	0.9379	0.9615	0.9495	0.9039	0.9452	4.37
Quad SA MSF CR ANMS	SA + MSF + CR + ANMS	0.9379	0.9615	0.9495	0.9039	0.9448	4.20
Triple SA CR SP	SA + CR + SP	0.9399	0.9592	0.9494	0.9037	0.9452	4.45
Dual SA MSF	SA + MSF	0.9384	0.9602	0.9492	0.9032	0.9438	4.29
Single CR	CR	0.9369	0.9612	0.9489	0.9028	0.9451	4.41
Single ANMS	ANMS	0.9341	0.9635	0.9486	0.9021	0.9464	4.31
Dual MSF ANMS	MSF + ANMS	0.9358	0.9618	0.9486	0.9023	0.9453	4.20
Single SP	SP	0.9372	0.9599	0.9484	0.9018	0.9463	4.44

#### Summary of key findings

In general, the results of the analysis show that (i) model performance is highly affected by the class imbalance and dense occlusion that are normally found in agricultural datasets taken in the real field, (ii) two-stage detector models are more stable and consistent in the current study under the above-mentioned difficult conditions, and (iii) the integration of carefully chosen modules is more important than stacking architectures in order to achieve effective crop and weed detection. In this regard, the SA-ANMS model presented in this study is a well-founded and practical approach that can provide stable minority-class detection and good generalization performance in difficult blackgram field conditions.

Beyond individual module performance, the ablation results reveal meaningful interaction effects between modules. SA and MSF exhibit partial redundancy: SA suppresses spatially homogeneous background at the backbone level, while MSF reintroduces global pooled features at the FPN level, diluting SA’s spatial specificity gains. Likewise, SA and CR move in opposite directions; SA decreases background activations within the feature extraction process, while CR increases the RoI features’ weights based on global context which might contain suppressed background signals, undoing part of SA’s work. These opposite transformations explain why the combinations of SA with CR or MSF obtain significantly worse performances compared to SA + ANMS despite increasing model complexity.

The peculiar results where the whole 5-module model perform significantly worse than the minimal SA + ANMS configuration are reported in. This is also in agreement with the phenomenon of feature interference when optimizing multiple modules: some individual helpful modules may cause detrimental gradients for joint optimization. For instance, SA (backbone), MSF (FPN) and CR (RoI) have contradicting goals at various network levels: SA seeks to suppress global background activations in order to strengthen spatial focus, while MSF and CR explicitly reuse global context signals. When these modules are trained jointly, their partially conflicting goals make the modules unable to achieve their full effect; furthermore, Shape Predictor makes additional regression-like objectives conflicting with ANMS’s run-time filtering function (training-inference mismatch).These findings suggest that for texturediscriminative agricultural detection tasks with moderate scale variation, a minimal coherent module set provides a stronger inductive bias than exhaustive module stacking.

## Future work

The future work will focus on extending the applicability of the SA-ANMS model for different crops by gradually increasing the dataset using the developed acquisition and annotation techniques for blackgram. The field images will be acquired from crops such as paddy and different pulses at similar growth stages with dense canopies and weed competition. The new dataset will help in retraining and testing the model without modifying the architecture of the SA-ANMS model, thus helping in analyzing the adaptability of the model to different crop types. In addition, the SA-ANMS model will be tested in cross-crop training settings, where the trained model on a different crop will be tested on another crop to analyze the generalization ability of the model. Further fine-tuning approaches, such as modifying the class-specific thresholds in Adaptive NMS and applying dataset-level balancing techniques, will also be explored since they can be applied without modifying the model complexity.

## Conclusion

This paper proposes an enhanced Faster R-CNN framework for accurate weed identification in real blackgram fields of Karnataka, India, to overcome difficult field conditions like dense crop canopy, high occlusion, large scale variations, and significant class imbalances, where the majority of weeds are less than 0.8% of the image area. In this paper, an extensive ablation study has been performed to systematically compare the performance of 32 different model variants, and the findings clearly show that selective module stacking, specifically the integration of Spatial Attention (SA) and Adaptive Non-Maximum Suppression (ANMS), provides the best possible trade-off between accurate weed detection (F1-score = 0.9547, Precision = 0.9439, Recall = 0.9658, mIoU = 0.9445) and efficiency (4.42 FPS) compared to models with full module stacking. As compared to other advanced models for object detection, like RetinaNet, EfficientDet, YOLOv5, and the baseline model Faster R-CNN, the proposed model SA-ANMS has demonstrated better performance on all counts. Even though one-stage object detection models like YOLOv5 are faster in terms of inference time, they have demonstrated lower precision and recall values in the presence of strong occlusion and small object dominance. The proposed model has demonstrated its capability to tackle these challenges by enhancing the ability to distinguish features using spatial attention and enhancing the post-processing step using class-aware suppression.

Further analysis reveals that the performance gains from modules such as Multi-Scale Fusion (MSF) and Shape-Aware Prediction (SP) are complementary in nature but can lead to redundancy and complexity if combined together indiscriminately.

The originality of this work is in the creation of a difficult real-world blackgram weed image dataset, the formulation of five enhancement modules specifically designed for small object detection in occluded and scaled images, and the ablation study framework that highlights both beneficial and detrimental effects of module interactions. This research work, as a whole, provides valuable insights into designing effective and accurate weed detection systems for precision agriculture tasks.

## Data Availability

The dataset used in this study will be made available upon reasonable request by contacting the corresponding author via email.
